# Selective depletion of a CD64-expressing phagocyte subset mediates protection against toxic kidney injury and failure

**DOI:** 10.1073/pnas.2022311118

**Published:** 2021-09-13

**Authors:** Natallia Salei, Xingqi Ji, Dalia Pakalniškytė, Vanessa Kuentzel, Stephan Rambichler, Na Li, Markus Moser, Katja Steiger, Thorsten Buch, Hans-Joachim Anders, Barbara U. Schraml

**Affiliations:** ^a^Walter-Brendel-Centre of Experimental Medicine, University Hospital, Ludwig-Maximilians-Universität in Munich, 82152 Planegg-Martinsried, Germany;; ^b^Biomedical Center, Institute for Cardiovascular Physiology and Pathophysiology, Faculty of Medicine, Ludwig-Maximilians-Universität in Munich, 82152 Planegg-Martinsried, Germany;; ^c^Division of Nephrology, The Seventh Affiliated Hospital, Sun Yat-sen University, 518107 Shenzhen, China;; ^d^Division of Nephrology, Medizinische Klinik und Poliklinik IV, University Hospital, Ludwig-Maximilians-Universität in Munich, 80366 Munich, Germany;; ^e^Institute of Experimental Hematology, School of Medicine, Technische Universität München, D-81675 Munich, Germany;; ^f^Institute of Pathology, School of Medicine, Technische Universität München, D-81675 München, Germany;; ^g^Institute of Laboratory Animal Science, University of Zürich, 8956 Zürich, Switzerland

**Keywords:** dendritic cells, macrophages, acute kidney injury, necroinflammation, cisplatin

## Abstract

Dendritic cells (DC), macrophages, and monocytes, collectively known as mononuclear phagocytes (MPs), critically control tissue homeostasis and immune defense. However, there is a paucity of models allowing to selectively manipulate subsets of these cells in specific tissues. The steady-state adult kidney contains four MP subsets with Clec9a-expression history that include the main conventional DC1 (cDC1) and cDC2 subtypes as well as two subsets marked by CD64 but varying levels of F4/80. How each of these MP subsets contributes to the different phases of acute kidney injury and repair is unknown. We created a mouse model with a Cre-inducible lox-STOP-lox-diphtheria toxin receptor cassette under control of the endogenous CD64 locus that allows for diphtheria toxin–mediated depletion of CD64-expressing MPs without affecting cDC1, cDC2, or other leukocytes in the kidney. Combined with specific depletion of cDC1 and cDC2, we revisited the role of MPs in cisplatin-induced kidney injury. We found that the intrinsic potency reported for CD11c^+^ cells to limit cisplatin toxicity is specifically attributed to CD64^+^ MPs, while cDC1 and cDC2 were dispensable. Thus, we report a mouse model allowing for selective depletion of a specific subset of renal MPs. Our findings in cisplatin-induced injury underscore the value of dissecting the functions of individual MP subsets in kidney disease, which may enable therapeutic targeting of specific immune components in the absence of general immunosuppression.

## Significance

There continues to be a paucity in models to target mononuclear phagocyte (MP) subsets selectively and in specific tissues. We report a mouse model allowing for selective depletion of a specific subset of kidney MPs that is based on a Cre-inducible lox-STOP-lox-diphtheria toxin receptor gene controlled by the endogenous CD64 promoter. Combined with specific targeting of conventional DC1 (cDC1) and cDC2, we revealed that CD64^+^ MPs account for the reported ability of CD11c^+^ cells to limit cisplatin nephrotoxicity, while cDC1 and cDC2 are dispensable. Our study highlights that individual MP subsets have unique functions in kidney pathology. Combined with various CRE drivers, CD64-lox-STOP-lox-diphtheria toxin receptor mice might be used to study CD64-expressing cells in other tissues.

Mononuclear phagocytes (MP) critically control barrier integrity, tissue homeostasis, and immune responses and include monocytes, macrophages, and dendritic cells (DCs) (1). Despite access to several ‘‘pan-macrophage’’ and “pan-DC” models for gene manipulation and cell depletion, there is a paucity in models to target macrophage and DC subsets selectively and in specific tissues (2, 3). Additionally, macrophages, monocytes, and DCs are notoriously difficult to distinguish based on surface markers, such as CD11c, which can be up- or down-regulated (1, 3, 4). As a result, the unique functions of MP subtypes in immunity, inflammation, wound repair, and specific tissues remain debated.

Most macrophages arise during embryogenesis, whereas conventional or classical DCs (cDCs), plasmacytoid DCs (pDCs), and monocytes arise from committed bone marrow progenitors (1, 2, 5). We have previously shown that precursors of cDCs in mice express the C type lectin receptor DNGR-1 (also known as CLEC9A, encoded by the *Clec9a* gene) (6). Although DNGR-1 is also expressed in differentiated type1 cDCs (cDC1) and, to a lower level, on pDCs, it is not expressed in other lymphoid and myeloid lineages, including precursors for monocytes, granulocytes, or lymphoid cells (6–8). By crossing *Clec9a*^*cre*^ mice to *Rosa*^*lox-stop-lox-*^yellow fluorescent protein (YFP) or *Rosa*^*lox-stop-lox-*^TOMATO mice (6, 9) we have demonstrated that the adult kidney contains four subsets of MPs with prominent *Clec9a*-expression history, indicative of cDC origin (6, 9). These include the main cDC1 and cDC2 subtypes as well as CD64-expressing CD11b^hi^ and F4/80^hi^ cells (6, 9). These subsets are phenotypically, functionally, and transcriptionally distinct, express CD11c, and are uniformly marked by MHC (major histocompatibility complex) II (6, 9). Although F4/80^hi^ MPs phenotypically and transcriptionally resemble embryonic-derived macrophages (9, 10) in adulthood they acquire prominent *Clec9a*-expression history (9). Because their affiliation as macrophages or DCs remains controversial (9–15), we henceforth refer to these cells as F4/80^hi^ MPs.

Cell depletion using “pan-macrophage” and “pan-DC” models that would affect multiple kidney MP subsets has shown that MPs are critically involved in kidney immune defense but also contribute to kidney injury and subsequent tissue repair (4, 13, 16, 17). Thus, defining the functions of specific subsets of MPs in kidney disease could help to dissect their contributions to kidney pathology and repair, which could subsequently be targeted for therapeutic purposes. Toxic acute kidney injury (AKI) is a common side effect resulting from cisplatin nephrotoxicity during cancer therapy (15). In mice, cisplatin treatment similarly induces nephrotoxicity, but the role of individual MP subsets in disease remains unclear. Preemptive depletion of CD11c^+^ cells with diphtheria toxin (DT) in mice in which the CD11c promoter drives expression of a DT receptor (DTR; *CD11c*^*DTR*^ mice) aggravates cisplatin-induced AKI (18). Because CD11c is not exclusively restricted to DCs but can also be found on some tissue macrophages as well as activated B and T cells, depletion in this model affects multiple cell types (1, 3). Notably, preemptive depletion of all phagocytic cells, including CD11c^+^ cells, using clodronate liposomes, does not alter the severity of cisplatin-induced AKI (19). These results seem conflicting when assuming that both models affect similar MPs. However, clodronate liposome treatment also depletes blood monocytes and both interventions may deplete multiple cell types with partially overlapping phenotype but distinct functions in toxic tissue injury. Thus, more specific models are needed to investigate the role of MP subsets in kidney disease.

Currently, tools for cell depletion do not accurately distinguish between the different types of kidney MPs. We hypothesized that specific deletion of the various subsets of kidney resident MPs before exposing mice to cisplatin would help to identify the MP subset that can mitigate nephrotoxic kidney injury and failure. We generated a mouse model with expression of a lox-STOP-lox-DTR construct under control of the endogenous *Cd64* locus. Through an intercross with *Clec9a*^*cre*^ mice, these mice allow for DT-mediated depletion of F4/80^hi^ MPs in the kidney, while cDC1, cDC2, and other leukocytes are not affected. Importantly, DT treatment did not affect CD64-expressing macrophages/monocyte-derived cells, DCs, or other leukocytes in various other organs. In combination with models to deplete cDC1 and cDC2, we demonstrate that the protective effect previously attributed to CD11c^+^ cells in cisplatin AKI is predominantly mediated by CD64 expressing MPs but independent of cDC1 and cDC2.

## Results

### MPs with *Clec9a*-Expression History in Cisplatin Nephrotoxicity.

**Fig. 1.**
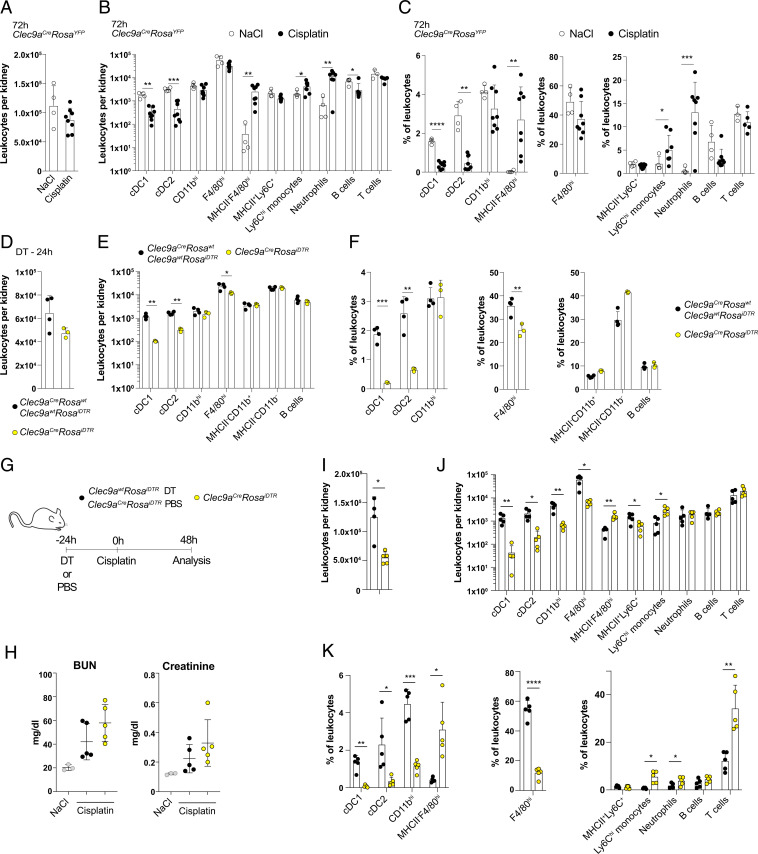
MPs with *Clec9a*-expression history in cisplatin nephrotoxicity. (*A*–*E*) *Clec9a*^*cre*^*Rosa*^*YFP*^ mice were injected with NaCl or cisplatin. A total 72 h later, renal leukocytes were analyzed by flow cytometry. Total leukocytes (*A*) as well as number (*B*) and frequency (*E*) of the indicated populations per kidney were calculated and plotted (*SI Appendix*, Fig. S1*B*). (*D*–*F*) *Clec9a*^*cre*^*Rosa*^*iDTR*^ mice were treated with PBS or DT, and littermate controls (*Clec9a*^*wt*^*Rosa*^*iDTR*^ or *Clec9a*^*cre*^*Rosa*^*wt*^) were injected with DT. A total 24 h later, total leukocytes (*D*) per kidney were quantified, and the number (*E*) and frequency (*F*) of the indicated populations per kidney were calculated. Data are combined from two independent experiments. (*G*–*K*) *Clec9a*^*cre*^*Rosa*^*iDTR*^ or *Clec9a*^*wt*^*Rosa*^*iDTR*^ mice were injected with DT followed by cisplatin treatment 24 h later. Mice were analyzed 48 h later. (*G*) Schematic representation of experimental design. (*H*) Serum creatinine and blood urea nitrogen (BUN) levels. (*I*–*K*) Kidneys were analyzed by flow cytometry. Total leukocytes (*I*) as well as number (*J*) and frequency (*K*) of the indicated populations per kidney were calculated and plotted. (*SI Appendix*, Fig. S1*B*) per kidney are shown. Data are representative of two independent experiments with *n* = 3 and similar results. Each dot represents one mouse. Horizontal bars represent mean, error bars represent SD, *P < 0.05, **P < 0.01, ***P < 0.001, and ****P < 0.0001.

We first investigated leukocyte dynamics in *Clec9a*^*cre*^*Rosa*^*YF*P^ mice with cisplatin nephrotoxicity to define the phenotype of MPs with *Clec9a*-expression history in toxic AKI. Cisplatin treatment of mice leads to acute tubular necrosis and kidney failure evident by a rapid increase in serum creatinine and urea levels after cisplatin exposure. We have shown that 3 d after cisplatin treatment, kidneys contain five populations with prominent *Clec9a-*expression history: cDC1, cDC2, CD11b^hi^, and F4/80^hi^ cells as well as a small population of MHCII^neg^F4/80^hi^ cells that arises from down-regulation of MHCII on F4/80^hi^ cells (9). Similarly, *Clec9a*-expression history was found in cDC1, cDC2, CD11b^hi^, and F4/80^hi^ cells 48 h after cisplatin treatment but MHCII^neg^F4/80^hi^ cells were barely detectable at this time (*SI Appendix*, Fig. S1 *A*–*C* and *F*). Notably, the frequency and number of cDC1 and cDC2 were reduced in kidneys from cisplatin-treated mice compared to controls 48 and 72 h after treatment, whereas frequency and number of F4/80^hi^ and CD11b^hi^ cells remained constant (Fig. 1 *A*–*C* and *SI Appendix*, Fig. S1 *B*–*F*). CD19^+^ B cells and CD3^+^ T cells remained unchanged, while monocytes (MHCII^−^CD11b^+^Ly6C^hi^ cells) and neutrophils (MHCII^−^CD11b^+^Ly6G^+^ cells) were increased as sign of necroinflammation (20). Thus, in addition to our previous observation that F4/80^hi^ cells alter their phenotype and transcriptional profile in response to cisplatin (9), we observed that cDC1 and cDC2 are lost from the inflamed kidney, leading us to hypothesize that MP subsets may play different roles in this disease model.

To assess the involvement of cells with *Clec9a*-expression history in cisplatin-induced AKI, we used *Clec9a-cre* mice crossed to *Rosa26*^*lox-STOP-lox-DTR*^ mice (*Clec9a*^*cre*^*Rosa*^*iDTR*^) (21, 22). A total 24 h after a single injection of DT, kidney cDC1 and cDC2 as well as F4/80^hi^ cells were efficiently depleted in *Clec9a*^*cre*^*Rosa*^*iDTR*^ mice, whereas CD11b^hi^ DCs and other leukocytes were not affected (Fig. 1 *D*–*F*). We next subjected *Clec9a*^*cre*^*Rosa*^*iDTR*^ mice and *Clec9a*^*wt*^*Rosa*^*iDTR*^ control mice to a single injection of DT and 24 h later treated the mice with cisplatin (Fig. 1 *G*–*K*). Several DT-treated *Clec9a*^*cre*^*Rosa*^*iDTR*^ mice showed a body weight loss exceeding 20% within 48 h after cisplatin treatment and thus reached experimental termination criteria. All mice were therefore analyzed 48 h after cisplatin treatment. cDCs and F4/80^hi^ MPs were efficiently depleted at this time point (Fig. 1 *I*–*K*), and we observed a trend for higher serum urea and creatinine levels in *Clec9a*^*cre*^*Rosa*^*iDTR*^ mice than in *Clec9a*^*wt*^*Rosa*^*iDTR*^ control mice, indicating more severe kidney failure (Fig. 1*H*). Of note, *CD11c*^*DTR*^ mice show higher mortality upon cisplatin exposure and have to be analyzed 48 h after disease induction (18). These data suggested that the protective effect previously attributed to CD11c^+^ leukocytes during cisplatin nephrotoxicity is mediated by MPs with *Clec9a-*expression history, leading us to investigate the cellular interplay of these MPs in more detail.

### Loss of cDC1 Does Not Influence the Severity of Cisplatin-Induced AKI.

**Fig. 2.**
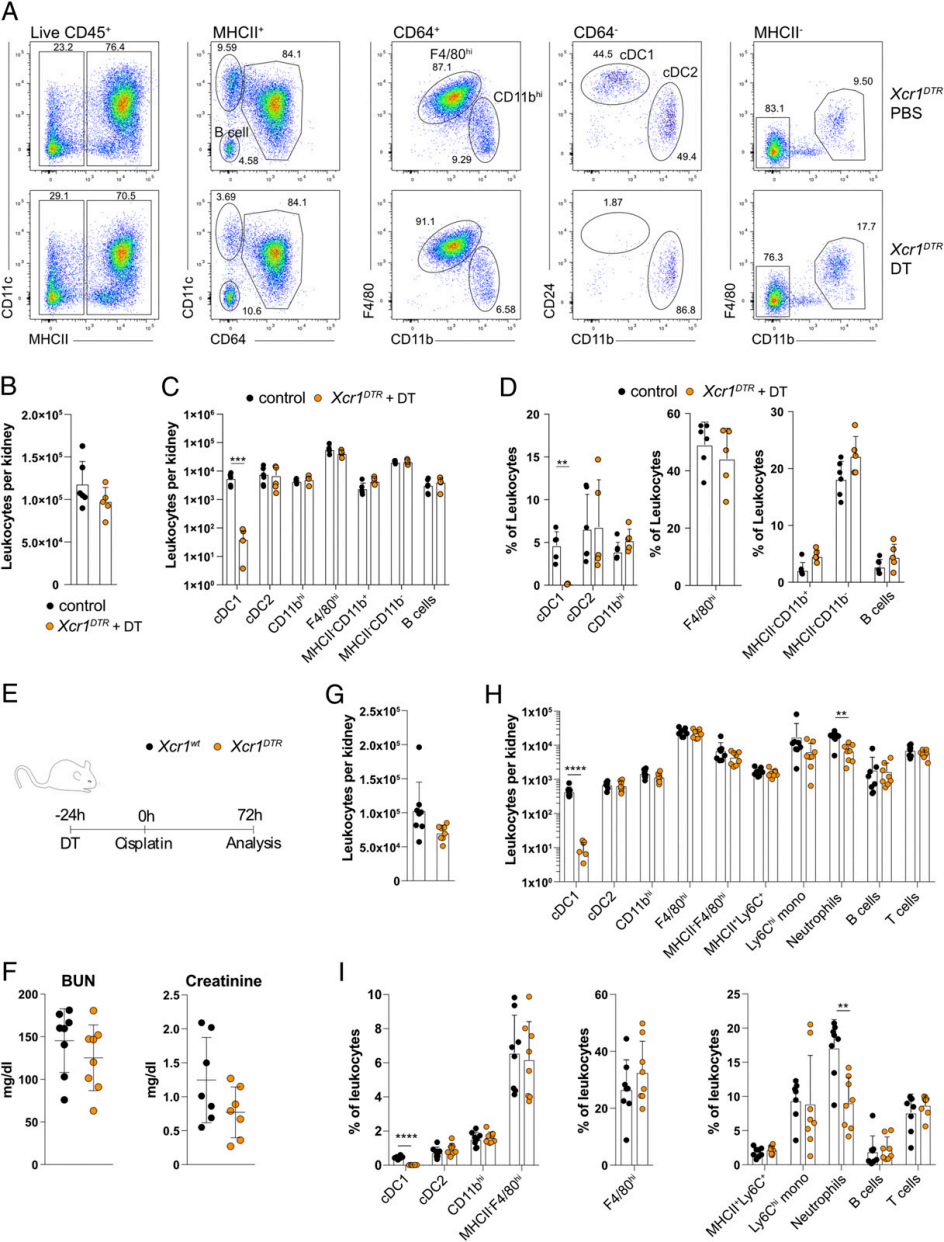
Loss of cDC1 does not influence susceptibility to cisplatin-induced AKI. (*A*–*D*) *Xcr1*^*DTR*^ mice were injected with PBS or DT, and 24 h later, kidney leukocytes were analyzed by flow cytometry. (*A*) Live CD45.2^+^ MHCII^+^ cells were gated and subdivided into CD11c^+^CD64^−^ and CD64^+^ cells as well as CD11c^−^CD64^−^ cells representing B cells. CD24^+^ cDC1 and CD11b^+^ cDC2 were identified in the CD11c^+^CD64^−^ fraction. CD64^+^ cells were further divided into F4/80^hi^ and CD11b^hi^ DCs. MHCII^−^ cells were subdivided into CD11b^+^ cells (entailing mostly neutrophils and monocytes) and CD11b^−^ cells (containing mostly T cells). Total leukocytes per kidney (*B*) as well as number (*C*) and frequency (*D*) of indicated populations per kidney are shown. Data are combined from two independent experiments. (*E*–*I*) *Xcr1*^*DTR*^ or littermate control mice were injected with DT followed by cisplatin treatment (15 mg/kg body weight [b. w.]) 24 h later. A total 72 h after cisplatin injection, mice were analyzed. (*E*) Schematic representation of experimental design. (*F*) Serum creatinine and BUN levels were quantified. Total leukocytes per kidney (*G*) as well as number (*H*) and frequency (*I*) of indicated populations per kidney are shown (Fig. 1*A*). Data are combined from two independent experiments. Each dot represents one mouse. Horizontal bars represent mean, error bars represent SD, **P < 0.01, and ****P < 0.0001.

To specifically deplete cDC1 in renal disease, we obtained *Xcr1*^*Venus-DTR*^ mice (*Xcr1*^*DTR*^). XCR-1 is a chemokine receptor specifically expressed by cDC1 across tissues, and *Xcr1*^*DTR*^ mice allow for efficient depletion of cDC1 in lymphoid tissues (23). Indeed, DT treatment of *Xcr1*^*DTR*^ mice efficiently and selectively depleted cDC1 from kidneys 24 h later, whereas other MP or leukocyte populations remained unaltered (Fig. 2 *A*–*D*). We next assessed the influence of cDC1 depletion on the severity of cisplatin-induced AKI. *Xcr1*^*DTR*^ and littermate control mice were treated with DT 24 h prior to injection with cisplatin. A total 72 h after cisplatin treatment, all mice showed clear signs of tubular necrosis and loss of kidney function, but serum urea and creatinine levels were similar between the two groups (Fig. 2*F*). A reduction of cDC1 in kidneys from DT- and cisplatin-treated *Xcr1*^*DTR*^ compared to control mice was confirmed by flow cytometry (Fig. 2 *G*–*I*), indicating that a single injection of DT is sufficient to deplete cDC1 for the course of the 3-d experiment. As expected, the frequency and number of other DC subsets was unaltered (Fig. 2 *H* and *I*). Notably, *Xcr1*^*DTR*^ mice showed reduced frequency and number of neutrophils (Fig. 2 *H* and *I*), suggesting that cDC1 may recruit neutrophils into the cisplatin-treated kidney although neutrophils are dispensable for the development for cisplatin AKI (20, 24). Taken together, these data indicate that selective loss of cDC1 does not influence disease severity in cisplatin-induced AKI.

### *Clec9a*^*cre*^*Irf4*^*fl/f*^^*l*^ Mice Do Not Show Altered Disease Severity in Cisplatin-Induced AKI.

**Fig. 3.**
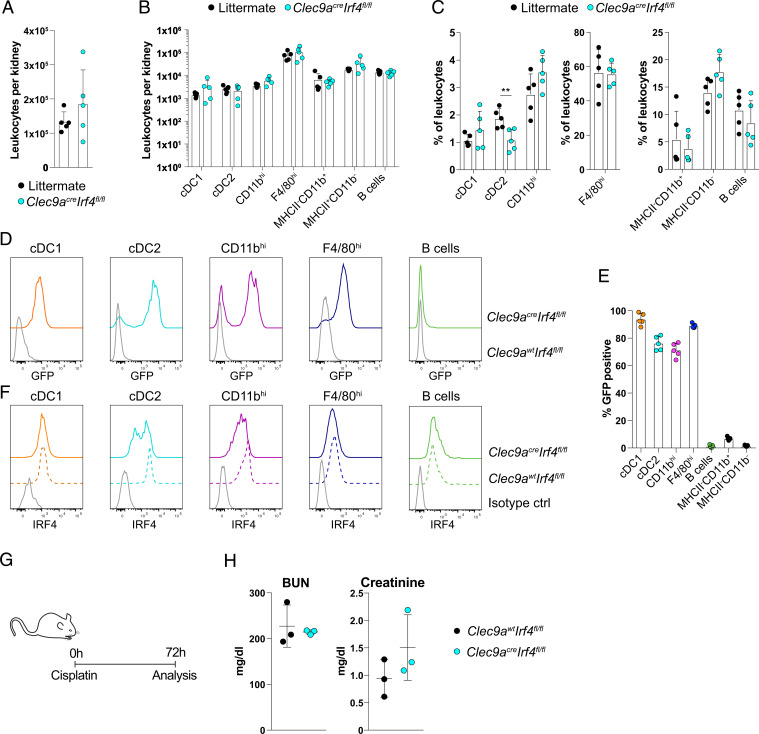
*Clec9a*^*cre*^*Irf4*^*fl/fl*^ mice do not show altered susceptibility to cisplatin-induced AKI. (*A*–*F*) Kidneys from *Clec9a*^*cre/cre*^*Irf4*^*fl/fl*^ or littermate control mice were analyzed by flow cytometry. Total kidney leukocytes (*A*) as well as number (*B*) and frequency (*C*) of the indicated populations per kidney (*SI Appendix*, Fig. S2) were calculated and plotted. (*D*–*F*) Kidney cDC1, cDC2, CD11b^hi^, and F4/80^hi^ MPs as well as B cells as specificity control were analyzed for GFP and IRF4 expression by flow cytometry. (*D*) GFP levels and (*E*) the frequency of GFP-positive cells within each population are shown to indicate excision of the floxed allele. Gray traces represent GFP fluorescence in littermate controls *(Clec9a*^*wt/wt*^*Irf4*^*fl/fl*^*)*. (*F*) IRF4 protein was revealed by intranuclear staining with an anti-IRF4 antibody. Gray traces represent staining with isotype-matched control antibody. (*G* and *H*) Cisplatin-induced AKI in *Clec9a*^*wt/wt*^*Irf4*^*fl/fl*^ and *Clec9a*^*cre/cre*^*Irf4*^*fl/fl*^ mice. (*G*) Schematic representation of experimental design. Mice were injected with 20 mg/kg b. w. cisplatin and analyzed 72 h later. (*H*) Serum creatinine and BUN levels are shown. Each dot represents one mouse. Data are representative of two independent experiments with *n* = 3 and similar results. Horizontal bars represent mean, error bars represent SD, and **P < 0.01.

Loss of *Irf4* inhibits cDC2 development in some organs and strongly impacts the function of the remaining cDC2 (25–31). Kidney cDC2 and CD11b^hi^ MPs express higher levels of interferon regulatory factor 4 (IRF4) compared to cDC1 and F4/80^hi^ MPs, in which IRF4 expression is low (9). To assess the role of cDC2 in cisplatin AKI, we therefore crossed *Clec9a*^*cre*^ mice to *Irf4-floxed* (*Irf4*^*fl/fl*^) mice (32). As expected (27), cDC2 in kidney and spleen of *Clec9a*^*cre*^*Irf4*^*fl/fl*^ mice were reduced compared to littermate controls, whereas other MPs or leukocytes were unaltered (Fig. 3 *A*–*C* and *SI Appendix*, Fig. S2). *Irf4*^*fl/fl*^ mice carry an enhanced green fluorescent protein (eGFP) reporter expressed upon Cre-mediated recombination of the loxp sites, allowing to trace cells with successful DNA recombination by eGFP fluorescence (32). As expected, recombination efficiency was high in the four MP subsets that show *Clec9a*-expression history but not in other leukocytes (Fig. 3 *D* and *E*), confirming specificity and efficiency of *Clec9a*^*cre*^-mediated DNA recombination. CD11b^hi^ DCs and the few remaining cDC2 in kidneys from *Clec9a*^*cre*^*Irf4*^*fl/fl*^ mice showed reduced levels of IRF4 protein compared to littermate controls (Fig. 3*F*), suggesting functional impairment of these cells (25–29). It is noteworthy that despite efficient excision of the floxed allele in *Clec9a*^*cre*^*Irf4*^*fl/fl*^ mice (Fig. 3 *D* and *E*), staining with the anti-IRF4 antibody was higher than with the isotype-matched control antibody. It is possible that this reflects residual IRF4 protein resulting from transcription though the internal stop of the GFP reporter (32), but it could also reflect IRF4 protein expression from an earlier state of cell development or nonspecific staining of the anti-IRF4 antibody compared to isotype-matched control antibody. A total 3 d after treatment of *Clec9a*^*cre*^*Irf4*^*fl/fl*^ mice and *Clec9a*^*wt*^*Irf4*^*fl/fl*^ littermate controls with cisplatin, serum creatinine and urea levels were similar between the two groups (Fig. 3 *G* and *H*). Thus, cDC2 play a negligible role in the induction of cisplatin-induced AKI. We therefore speculated that the protective effect previously attributed to CD11c^+^ cells in cisplatin-induced renal injury must be mediated by CD64 expressing MPs.

### *Clec9a*^*cre*^*Cd64*^*iDTR*^ Mice Allow to Deplete F4/80^hi^ MPs from Kidneys.

**Fig. 4.**
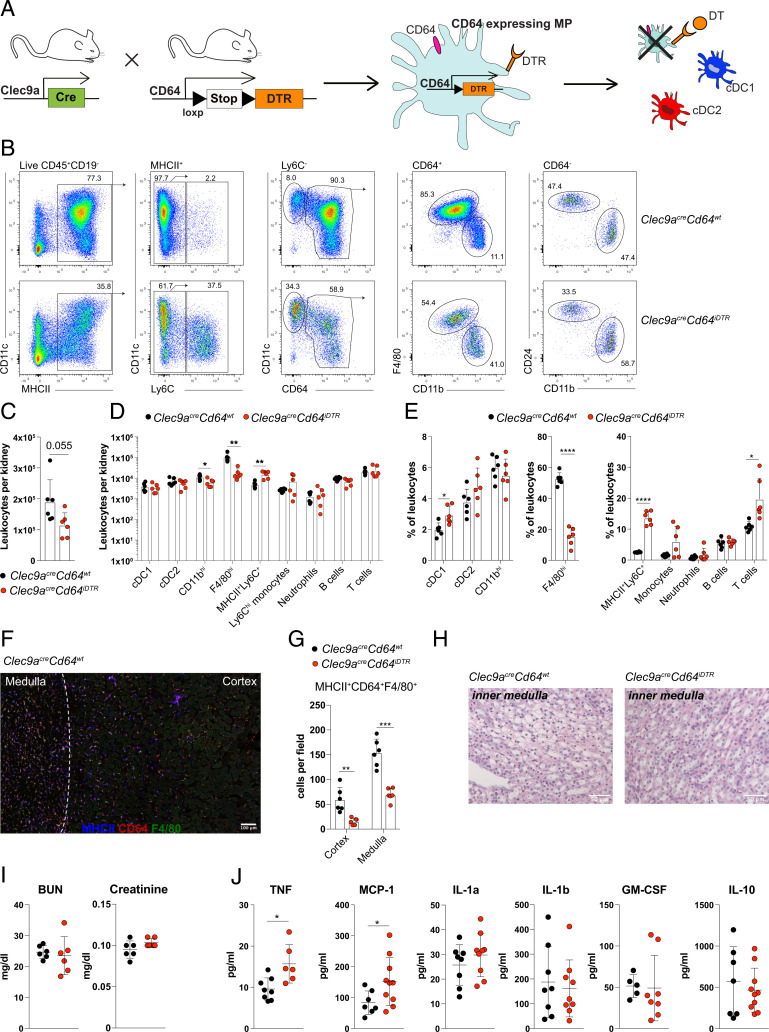
*Clec9a*^*cre*^*Cd64*^*iDTR*^ mice allow for specific depletion of renal F4/80^hi^ MPs. (*A*) Schematic representation of cell depletion in *Clec9a*^*cre*^*Cd64*^*iDTR*^ mice. (*B*–*H*) *Clec9a*^*wt/cre*^*CD64*^*iDTR*^ and *Clec9a*^*wt/cre*^*Cd64*^*wt*^ mice were injected with DT and analyzed 24 h later. (*B*) Kidney leukocytes were analyzed by flow cytometry. Live CD45.2^+^MHCII^+^ cells were gated and divided into Ly6C^+^ and Ly6C^−^ cells, as indicated. Ly6C^−^ cells were further divided into CD11c^+^CD64^−^ and CD64^+^ cells, and within the CD11c^+^CD64^−^ fraction, CD24^+^ cDC1 and CD11b^+^ cDC2 were gated. CD64^+^ cells were divided into F4/80^hi^ and CD11b^hi^ DCs. (*C*) Total kidney leukocytes as well as number (*D*) and frequency (*E*) of the indicated populations per kidney are shown. (*F* and *G*) Kidney cryosections from DT-treated *Clec9a*^*wt/cre*^*Cd64*^*iDTR*^ and *Clec9a*^*wt/cre*^*Cd64*^*wt*^ mice were stained for MHCII (blue), CD64 (red), and F4/80 (green). MHCII^+^CD64^+^F4/80^+^ cells were identified, and the number of these cells per field in renal cortex and medulla were quantified and plotted. (Scale bar: 100 μm.) The average number of cells per six fields of view is shown (*G*). (*H*) Normal kidney architecture and function in DT-treated *Clec9a*^*wt/cre*^*CD64*^*iDTR*^ mice. Representative PAS staining of renal medulla 24 h after DT treatment is shown. (Scale bar: 50 μm.) (*I*) Serum creatinine and BUN levels 24 h after DT treatment. (*J*) Serum levels of indicted cytokines 24 h after DT treatment. Each dot represents one mouse. Horizontal bars represent mean, error bars represent SD, *P < 0.05, **P < 0.01, and ****P < 0.0001. Data from are combined from at least two independent experiments.

To specifically deplete CD64^+^ MPs from kidneys, we constructed a mouse model in which the endogenous *Cd64* locus drives expression of a lox-STOP-lox-DTR construct (*SI Appendix*, Fig. S3*A*). In these mice, DTR expression is expected in cells with active transcription of the *Cd64* locus but only when the loxp sites were previously excised. Crossing these mice to *Clec9a*^*cre*^ mice would therefore lead to specific expression of DTR on CD64-expressing cells with *Clec9a*^*cre*^ expression history, allowing for specific deletion of kidney CD11b^hi^ and F4/80^hi^ MPs (Fig. 4*A*). Successful generation of *Cd64*^*iDTR*^ mice was confirmed using Southern Blot (*SI Appendix*, Fig. S3*B*). To circumvent inflammatory defects associated with loss of CD64, we inserted the DTR transgene using a self-cleaving 2A sequence leaving CD64 intact, as confirmed by flow cytometry (*SI Appendix*, Fig. S3*C*). In *Clec9a*^*cre*^*Cd64*^*iDTR*^ mice, we detected unimodal DTR expression on renal F4/80^hi^ MPs to a lower extent on CD11b^hi^ MPs but not on cDC1 and cDC2 or monocytes (*SI Appendix*, Fig. S3*D*), as expected. Accordingly, a single injection of DT in *Clec9a*^*cre*^*Cd64*^*iDTR*^ mice lead to efficient depletion of F4/80^hi^ MPs (sixfold reduction, Fig. 4 *D* and *E*) from kidneys 24 h later without affecting cDC1, cDC2, or other leukocytes, including Ly6C^hi^ monocytes, neutrophils, B cells, and T cells (Fig. 4 *B*–*D* and *SI Appendix*, Fig. S3*E*). Notably, treatment of *Clec9a*^*cre*^*Cd64*^*iDTR*^ mice with DT induced a minor loss of CD11b^hi^ MPs, despite the fact that these cells express CD64 and have *Clec9a*-expression history (9). This indicates a reduced sensitivity of CD11b^hi^ MPs to DT treatment, consistent with low DTR expression, which is likely a consequence of low CD64 expression (*SI Appendix*, Fig. S3 *C* and *D*). Notably, DT treatment of *Clec9a*^*cre*^*Cd64*^*iDTR*^ mice caused a significant increase in MHCII^+^Ly6C^+^ cells (Fig. 4 *B*–*E*), that also expressed CD11b and CD64 and could thus constitute tissue-infiltrating monocytes (33, 34). Loss of F4/80^hi^ MPs was also confirmed by microscopy, which revealed a fourfold reduction of F4/80^hi^ cells in kidney cortex and a 2.2-fold reduction in medulla of DT-treated *Clec9a*^*cre*^*Cd64*^*iDTR*^ mice (Fig. 4 *F* and *G*).

We additionally performed depletion analysis in *Clec9a*^*cre*^*Cd64*^*iDTR*^ mice across various lymphoid and nonlymphoid tissues. DT injection in *Clec9a*^*cre*^*Cd64*^*iDTR*^ mice did not alter frequency or number of cDC1, cDC2, and CD64^+^ cells, which include various types of macrophages and monocytes in spleen, skin, lung, brain, and liver when compared to DT-treated controls (*SI Appendix*, Figs. S4 and S5). Other leukocytes were also unaltered (*SI Appendix*, Figs. S4 and S5). We did not observe signs of systemic neutrophilia or monocytosis (35) apart from a slight increase of neutrophils in liver of *Clec9a*^*cre*^*Cd64*^*iDTR*^ mice (*SI Appendix*, Figs. S4 and S5). Serum levels of TNF and CCL2 (MCP-1), which promote monocyte recruitment, were increased in DT-treated *Clec9a*^*cre*^*Cd64*^*iDTR*^ compared to control mice (Fig. 4*J*). Thus, *Clec9a*^*cre*^*Cd64*^*iDTR*^ mice allow for selective depletion of CD64^+^ cells with *Clec9a*-expression history in kidney but do not affect cDC1, cDC2, or other CD64^+^ cells across lymphoid and nonlymphoid organs.

Despite depleting a major population of leukocytes from the kidney during homeostasis, DT treatment of *Clec9a*^*cre*^*Cd64*^*iDTR*^ mice did not cause significant alterations of serum urea and creatinine levels (Fig. 4*I*), indicating normal excretory kidney function. DT-treated *Clec9a*^*cre*^*Cd64*^*iDTR*^ mice showed similar kidney architecture to DT-treated littermates as judged by Periodic Acid Schiff (PAS) staining (Fig. 4*H* and *SI Appendix*, Fig. S3*F*). Thus, DT treatment of *Clec9a*^*cre*^*Cd64*^*iDTR*^ mice selectively depletes renal F4/80^hi^ MPs and to a lower extent CD11b^hi^ DCs without affecting kidney function and architecture. Taken together, *Clec9a*^*cre*^*Cd64*^*iDTR*^ mice can be used to specifically assess the role of F4/80^hi^ MPs in kidney injury.

### Loss of F4/80^hi^ MPs Increases Susceptibility to Cisplatin-Induced Renal Injury.

**Fig. 5.**
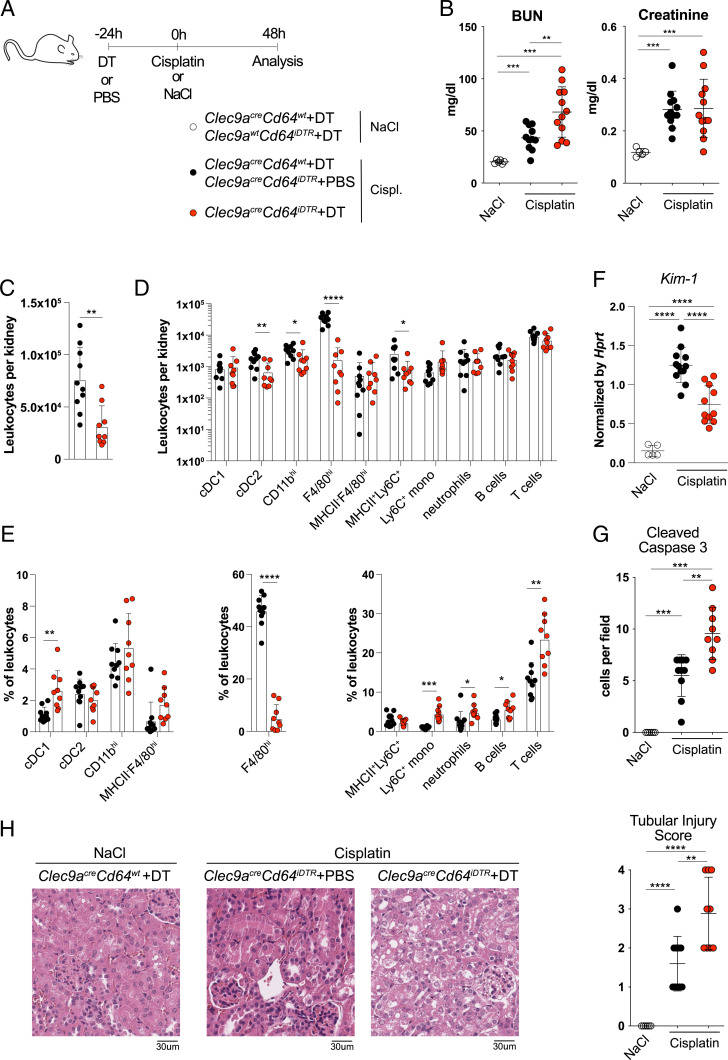
Loss of F4/80^hi^ MPs increases susceptibility to cisplatin-induced AKI. (*A*–*G*) Cisplatin-induced AKI in *Clec9a*^*cre*^*Cd64*^*iDTR*^ mice. (*A*) Experimental design. *Clec9a*^*cre*^*Cd64*^*iDTR*^ mice were injected with DT to induce cell depletion. As controls for cell depletion and DT treatment, *Clec9a*^*cre*^*Cd64*^*iDTR*^ and *Clec9a*^*cre*^*Cd64*^*wt*^ mice were injected with PBS or DT, respectively. Control mice were treated with either NaCl or cisplatin to induce AKI 24 h later, as indicated. Cisplatin/NaCl injected mice were analyzed 48 h later. (*B*) BUN and serum creatinine levels are shown. (*C*–*E*) Kidneys from mice were analyzed 48 h after cisplatin treatment by flow cytometry. Total leukocytes per kidney (*C*) as well as number (*D*) and frequency (*E*) of the indicated populations per kidney was calculated. (*F*) mRNA from kidney was isolated and analyzed for expression of *Kim*-1. Expression was normalized to *Hprt*. (*G*) Kidneys were analyzed for cleaved Caspase-3 by immunohistochemistry. The average number of cleaved Caspase-3–positive tubular epithelial cells per 10 high power fields of view was calculated. (*H*) Representative histology of kidney cortex by hematoxylin and eosin staining and histopathological score of tubular damage. (Scale bar: 30 μm.) Each dot represents one mouse. Horizontal bars represent mean, error bars represent SD, *P < 0.05, **P < 0.01, ***P < 0.001, ****P < 0.0001, and only statistically significant differences are indicated. Data are combined from three independent experiments.

To address the role of F4/80^hi^ MPs in cisplatin-induced AKI, we injected *Clec9a*^*cre*^*Cd64*^*iDTR*^ mice with DT (Fig. 5*A*). As controls, we used cohorts of *Clec9a*^*cre*^*Cd64*^*iDTR*^ and *Clec9a*^*cre*^*Cd64*^*wt*^ mice that were injected with phosphate buffered saline (PBS) or DT, respectively. Mice were treated with cisplatin or NaCl as control 24 h later (Fig. 5*A*). Mice were analyzed 48 h after cisplatin treatment because weight loss of many DT-treated *Clec9a*^*cre*^*Cd64*^*iDTR*^ mice had reached 20% at this point, necessitating termination of the experiment. Analysis of kidney leukocytes showed a reduction of total leukocytes per kidney in *Clec9a*^*cre*^*Cd64*^*iDTR*^ mice, which predominantly reflected the loss of F4/80^hi^ MPs (Fig. 5 *C*–*E*). We also observed a twofold reduction of CD11b^hi^ DCs and cDC2 in kidneys from DT-treated *Clec9a*^*cre*^*Cd64*^*iDTR*^ mice compared to controls (Fig. 5 *D* and *E*). Importantly, compared to NaCl treatment, cisplatin did not induce CD64 expression on immune cell populations that normally do not express it, although higher levels of CD64 were observed on monocytes, F4/80^hi^, and CD11b^hi^ MPs (*SI Appendix*, Fig. S6*A*). These data indicate that the intersection of *Clec9a*^*Cre*^ and *CD64*^*iDTR*^ remains restricted to F4/80^hi^ and CD11b^hi^ MPs (*SI Appendix*, Fig. S6*A*). Thus, a single DT treatment is sufficient to deplete F4/80^hi^ MPs for the course of this 3-d experiment and that cells resembling F4/80^hi^ MPs are not generated by compensatory mechanisms of emergency hematopoiesis, such as monocyte differentiation. Despite alterations in frequencies, the total numbers of monocytes, neutrophils, and B and T cells per kidney were not affected (Fig. 5*D*). No differences were found in splenic DCs, macrophages, monocytes, neutrophils, and B and T cells between the two groups, indicating that depletion in *Clec9a*^*cre*^*Cd64*^*iDTR*^ mice does not cause systemic neutrophilia, as observed in other DC depletion models (*SI Appendix*, Fig. S6*B*). MHCII^+^Ly6C^+^ cells resembling monocytes were found in kidneys from both *Clec9a*^*cre*^*Cd64*^*iDTR*^ and control mice after cisplatin treatment (Fig. 5*D*). Serum creatinine and urea levels were significantly increased in cisplatin- compared to NaCl-treated mice, confirming induction of kidney damage and loss of tubular function (Fig. 5*B*). Induction of kidney damage was further confirmed by measuring expression of kidney injury molecule 1 (also known as T cell Ig and Mucin-containing 1/TIM-1), which was significantly increased in cisplatin compared to NaCl treatment (Fig. 5*F*). Serum urea was higher in *Clec9a*^*cre*^*Cd64*^*iDTR*^ mice compared to control-treated littermates 48 h after cisplatin treatment, suggesting increased susceptibility to disease (Fig. 5*B*), whereas creatinine levels showed no difference between the two groups at this time (Fig. 5*B*). In line with increased serum urea in DT-treated *Clec9a*^*cre*^*Cd64*^*iDTR*^ mice, these mice also showed increased apoptosis of renal tubular cells, as assessed by cleaved Caspase 3 staining (Fig. 5*G*) and more severe tubular damage (Fig. 5*H*). In conclusion, loss of F4/80^hi^ MPs leads to more pronounced cisplatin AKI.

## Discussion

Kidney MP subtypes exhibit a prominent phenotypic overlap, and these cells have predominately been studied as a functionally homogeneous entity. In the present study, we had hypothesized that specific deletion of MP subsets before exposing mice to cisplatin would help to identify the subtype that can mitigate nephrotoxic kidney injury and failure. Indeed, the generation of a *CD64*^*lox-STOP-lox*^*-DTR* mouse model allowed us to selectively deplete a subtype of CD64-expressing MPs but not cDC1, cDC2, or other leukocytes in the kidney. Importantly, CD64-expressing leukocytes in spleen, liver, lung, skin, and brain were unaffected by DT treatment of *Clec9a*^*cre*^*Cd64*^*iDTR*^ mice. In combination with models to specifically deplete cDC1 and cDC2, we could demonstrate that kidney CD64^+^ MPs but not cDC1 or cDC2 attenuate kidney injury upon cisplatin exposure. *Clec9a*^*cre*^*Cd64*^*iDTR*^ mice provide a powerful model to study the specific functions of F4/80^hi^ MPs in kidney pathology. Importantly, combined with macrophage- or monocyte-specific CRE drivers, *Cd64*^*iDTR*^ mice may also serve as a valuable tool to study the functions of other CD64-expressing leukocytes in immunity.

cDC1, though best known for their ability to promote cytotoxic T cell and Th1 responses (36–38), also influence inflammatory processes by controlling neutrophils (39–41). Selective depletion of cDC1 in *Xcr1*^*DTR*^ mice prior to cisplatin treatment did not alter serum creatinine and urea levels, demonstrating that these cells do not influence severity of cisplatin-induced AKI. This is possibly to be expected because few cDC1 remain in kidneys 3 d after cisplatin treatment. Notably, neutrophils were reduced in kidneys from DT-treated *Xcr1*^*DTR*^ mice after cisplatin exposure, suggesting that cDC1 may regulate neutrophil survival or recruitment into the kidney (39–41). Although neutrophils are dispensable in cisplatin AKI, they contribute to the pathology of kidney ischemia reperfusion injury (20, 24, 42). Thus, cDC1 may contribute to necroinflammation in situations when neutrophils contribute to pathology.

Loss of IRF4 inhibits cDC2 development in some organs and strongly impacts cDC2 function (25–31); we crossed *Clec9a*^*cre*^ mice to *Irf4*^*fl/fl*^ mice. Because cDC2 and CD11b^hi^ cells in kidneys express high levels of IRF4 (9), we expected to generate mice lacking functional cDC2 and CD11b^hi^ cells. As observed when using *CD11c*^*cre*^ as driver to delete IRF4 (27), *Clec9a*^*cre*^*Irf4*^*fl/fl*^ mice exhibited a modest reduction of cDC2 in steady-state kidneys. In the remaining cDC2 and in CD11b^hi^ DCs, IRF4 protein expression was decreased, as expected, suggesting functional impairment (25–31). *Clec9a*^*cre*^*Irf4*^*fl/fl*^ mice did not show altered susceptibility to cisplatin-induced AKI, indicating that cDC2 and possibly CD11b^hi^ cells do not mitigate nephrotoxic kidney injury. Constitutive lack of IRF4 leads to increased tubular necrosis, neutrophil influx, and inflammatory cytokine expression 24 h after renal artery clamping (43). Although the cellular mediator of IRF4 action in this model is unclear, a CD11c^+^ population is involved (43). Because cDC2 are the CD11c^+^ cell type in kidney expressing highest levels of IRF4 (9), it is likely that IRF4 acts in these cells. This can be addressed in *Clec9a*^*cre*^*Irf4*^*fl/fl*^ mice independent of secondary effects of loss of IRF4 in other immune populations, such as B or T cells.

To be able to address the specific functions of renal CD64-expressing DCs in immunity and kidney pathology, we generated a mouse model, in which the endogenous CD64 locus drives expression of a lox-STOP-lox-DTR construct. To circumvent inflammatory defects associated with loss of CD64 (44, 45), we inserted the *DTR* transgene using a self-cleaving 2A sequence behind the full-length *Cd64* coding sequence, leaving CD64 intact. By crossing *Clec9a*^*cre*^ to *Cd64*^*iDTR*^ mice, we expected to gain DTR expression on cells in which CD64 is actively transcribed and which show evidence of *Clec9a*-expression history, rendering these cells susceptible to DT treatment. Indeed, DT treatment of *Clec9a*^*cre*^*Cd64*^*iDTR*^ mice specifically and efficiently depleted CD64-expressing DCs from the kidney, while cDC1, cDC2, and other leukocytes were unaffected. CD64-expressing cells in other organs, which constitute mainly macrophages or monocyte-derived cells (15) were also unaffected. Importantly, F4/80^hi^ cells showed a stronger reduction following DT treatment than CD11b^hi^ DCs, despite the fact that CD11b^hi^ cells also exhibit *Clec9a*^*cre*^ expression history and express CD64. We attribute this reduced susceptibility of CD11b^hi^ DCs to DT treatment to lower DTR expression. Interestingly, the efficiency of F4/80^hi^ MP depletion was increased following cisplatin treatment compared to steady state, which could be related to a loss in kidney architecture during nephrotoxicity. After cisplatin treatment, kidney cDC2 numbers were twofold lower in DT-treated *Clec9a*^*Cre*^*CD64*^*iDTR*^ mice than in control mice. Because cisplatin causes a 10-fold reduction of kidney cDC1 and cDC2 over NaCl treatment within 48 h, we believe these differences to be a consequence of inflammatory changes associated with disease rather than an indicator of nonspecific cell depletion. Whether cisplatin induces cDC1/cDC2 death or whether these cells actively migrate out of the organ is unclear. Taken together, *Clec9a*^*cre*^*Cd64*^*iDTR*^ mice specifically deplete renal F4/80^hi^ MPs. Because CD64 is also induced on some DCs during infection with listeria and respiratory viruses (46, 47), *Clec9a*^*cre*^*Cd64*^*iDTR*^ mice will provide a useful tool to study the specific functions of inflammatory DCs.

DT-mediated depletion of CD11c^+^ cells can induce functional neutrophilia that influences immune responses in the kidney (35). A total 24 h after DT treatment, *Clec9a*^*cre*^*Cd64*^*iDTR*^ mice did not show infiltration of neutrophils into the kidney or other organs, barring a small increase of monocytes and neutrophils in liver. Following DT treatment, *Clec9a*^*cre*^*Cd64*^*iDTR*^ mice exhibited normal excretory kidney function and architecture, validating their use to study the role of F4/80^hi^ cells in kidney injury. Following depletion of F4/80^hi^ cells, we observed a significant increase in MHCII^+^Ly6C^+^ cells that also expressed CD64 and CD11b and therefore resembled monocytes, which acquire MHCII expression upon differentiation in tissues (15, 48). It is possible that these MHCII^+^Ly6C^+^ cells constitute monocytes attempting to fill the niche of F4/80^hi^ cells in the kidney, similar to monocytes replenishing the niche of Kupffer cells following depletion (33, 49). However, F4/80^hi^ MPs remain strongly reduced during cisplatin AKI even 3 d after DT treatment, suggesting that if replenishment of the cells by monocytes takes place, it is inefficient. Rather, MHCII^+^Ly6C^+^ cells may constitute a distinct subtype of inflammatory monocytes, as reported to appear in spleen after depletion of CD11c^+^ cells (34).

Exposing DT-treated *Clec9a*^*cre*^*CD64*^*iDTR*^ mice to cisplatin leads to more severe kidney damage and loss of excretory function, allowing to specifically attribute the protective effect previously reported for CD11c^+^ cells in cisplatin-induced AKI to the function of kidney F4/80^hi^ MPs. The fact that serum urea but not creatinine levels were increased in DT-treated *Clec9a*^*cre*^*Cd64*^*iDTR*^ mice could be related to the time point of analysis. Overall, kidney damage was mild 48 h after cisplatin exposure, and serum urea is a more sensitive biomarker of renal excretory function in mice than creatinine. In seeming paradox to the increased susceptibility of DT-treated *Clec9a*^*cre*^*Cd64*^*iDTR*^ mice to cisplatin nephrotoxicity, these mice showed lower expression of the tubular damage marker *Kim-1/Tim-1* compared to control mice. However, *Kim-1/Tim-1* serves as a receptor for phosphatidylserine exposed on apoptotic cells (50–52), and in cisplatin-induced AKI, *Kim-1/Tim-1* dampens inflammation by inhibiting inflammatory cytokine production from epithelial cells (53). These data suggest that F4/80^hi^ cells may ameliorate cisplatin-induced renal injury in part through interacting with renal tubular cells and by reducing their inflammatory properties.

Cisplatin is a commonly used chemotherapeutic agent with a major limiting factor: cytotoxic side effects on normal tissues, including tubular necrosis, leading to loss of kidney function as well as neurotoxicity and damage of the gastrointestinal epithelium (54–56). Cisplatin additionally induces loss of adipose tissue (57). In our experiments, cisplatin induced weight loss in all mice, but *Clec9a*^*Cre*^*CD64*^*iDTR*^ mice reached a critical weight loss defined by our animal license earlier, necessitating termination of the experiments after 48 h. This phenotype is reminiscent of the higher mortality reported for DT-treated *CD11c*^*DTR*^ mice (18). Because weight loss in cisplatin AKI relates to side effects on other tissues, adverse effects of cell depletion in *CD11c*^*DTR*^ and *Clec9a*^*Cre*^*CD64*^*iDTR*^ mice on tissue integrity and cisplatin-induced pathology in other organs will have to be investigated in future studies. In terms of kidney pathology, our studies demonstrate that distinct MP subsets have specific roles in renal disease. An increased understanding of the specific functions of DC and macrophage subsets in kidney pathology will pave the way to design therapeutic approaches to specifically target individual immune components during kidney injury and disease.

## Materials and Methods

### Mice.

*Clec9a*^*tm2.1(icre)Crs*^*(Clec9a*^*cre*^*)* (6), Gt(ROSA)26Sor^tm1(EYFP)Cos^ (*Rosa26*^*YFP*^*)* (58), Gt(ROSA)26Sor^tm1(HBEGF)Awai^ (*Rosa26*^*iDTR*^*)* (59), *Irf4-flox* (32), *Xcr1-venus-DTR* (23), *Cd64-lox-STOP-lox-DTR*, and C57BL/6J mice were bred at the Biomedical Center of Ludwig Maximilian University of Munich in specific pathogen-free conditions. Mice at the age of 8 to 14 wk were used for experiments. All animal procedures were performed in accordance with national and institutional guidelines for animal welfare and approved by the Regierung of Oberbayern.

### Cloning of CD64 Targeting Vector and Generation of *Cd64-lox-STOP-lox-DTR* Mice and Immunofluorescence Microscopy.

Refer to *SI Appendix*.

### Southern Blot.

Southern blot was performed using the Digoxigenin system for labeling and detection of nucleic acid (Roche Applied Science) according to the manufacturer’s instructions. PsiI and KpnI-HF restriction enzymes were used. The probe1 located downstream of the 3′ homology arm was generated using primers 5′ACT​TCG​CCC​CAA​AGT​CCT​AT and 5′TCT​CGC​TTA​CTT​GAG-​CAG​CA. Neo probe located in the Neo cassette was generated using primers 5′AAT​ATC​ACG​GGT​AGC​CAA​CG and 5′CAT​TGA​ACA​AGA​TGG​ATT​GCA​CGC.

### Cell Isolation.

Organs were isolated after perfusion with ice-cold PBS. Kidneys, lungs, and brains were cut into small pieces and digested in 2 mL of RPMI medium (Thermo Fisher Scientific) with 200 U/mL collagenase IV (Worthington) and 0.2 mg/mL DNase I (Roche) for 1 h at 37 °C with constant shaking (120 rpm). After digestion, cells were passed through a 70-µm strainer and washed with cold fluorescence-activated cell sorting (FACS) buffer (PBS [Sigma-Aldrich] containing 1% fetal bovine serum [FBS] [Sigma-Aldrich], 2.5 mM ethylenediaminetetraacetic acid [Invitrogen], and 0.02% sodium azide [Sigma-Aldrich]). Leukocytes were enriched with 70–37–30% Percoll gradient by centrifugation (935 × *g* for 30 min at room temperature), collected at the 70–37% interface, washed once, and resuspended in FACS buffer for analysis. Isotonic Percoll was made by adding 9 parts of Percoll (Sigma-Aldrich) to 1 part of 10× concentrated PBS.

Spleen was minced and digested in 1 mL of RPMI for 30 min as above. Erythrocytes were lysed with ammonium-chloride-potassium lysing buffer for 2 min on ice, washed, and resuspended in FACS buffer for analysis.

Liver was digested in 2 mL PBS containing Mg^2+^ and Ca^2+^ (Sigma-Aldrich, Cat. D8662) with 1 mg/mL collagenase IV, 60 U/mL DNase I, 2.4 mg/mL dispase II (Roche), and 3% FBS (Sigma-Aldrich) for 30 min at 37 °C with constant shaking (120 rpm). Cells were passed through a 100-µm strainer and centrifuged for 3 min at 50 × *g* and 4 °C to pellet hepatocytes. A supernatant containing liver leukocytes was washed with FACS buffer (320 × *g*, 7 min, 4 °C) and resuspended in FACS buffer for analysis.

For skin isolation, mouse ears were split into dorsal and ventral parts and placed on PBS containing 2.5 mg/mL dispase II for 2 h at 37 °C or overnight at 4 °C. Epidermis and dermis were then separated and digested in 2 mL RPMI with 200 U/mL collagenase IV and 0.2 mg/mL DNase I for 1 h at 37 °C with constant shaking (200 rpm). After digestion, cells were passed through a 70-µm strainer, washed with cold FACS buffer, and enriched using a Percoll gradient as described above.

### Flow Cytometry.

For surface staining, cells were incubated in 50 μL FACS buffer with purified anti-mouse CD16/32/FcBlock for 10 min at 4 °C, then additional antibodies were added and incubated for 20 min at 4 °C. After staining, cells were washed twice and resuspended in FACS buffer for analysis. Dead cells were identified with Dapi (Sigma-Aldrich). For intracellular staining, cells were first stained with antibody against extracellular epitopes and then fixed with 2% paraformaldehyde at room temperature for 15 min. Intranuclear staining was performed by using Foxp3 transcription factor staining set (Thermo Fisher Scientific) according to the manufacturer’s protocol. Dead cells were identified with Fixable Viability Dye eFluor 780 (Thermo Fisher Scientific).

Flow cytometry was performed on an LSR Fortessa (BD Biosciences) with subsequent data analysis using FlowJo software (Tree Star, Inc.). Cells were quantified by using CountBright Absolute Counting Beads (Thermo Fisher Scientific).

The following antibodies were purchased from Biolegend: anti–CD45.2-R-phycoerythrin-cyanine 7 (PECy7) (clone 104), anti–MHCII I-A/I-E-alexa fluor (AF) 700 (clone M5/114.15.2), anti–CD11b-Brilliant Violet (BV) 421 (clone M1/70), anti–CD11c-BV786 (N418), anti–CD11c-BV421 (clone N418), anti–F4/80-BV786 (clone BM8), anti–F4/80-AF647 (clone BM8), anti–CD24-BV605 (clone M1/69), anti–XCR1-BV421 (clone ZET), anti–CD64-R-phycoerythrin (PE) (clone ×54-5/7.1), anti–CD64-allophycocyanin (APC) (clone ×54-5/7.1), anti–IRF4-AF647 (clone IRF42EA), anti–Ly6C-PerCP-Cy5.5 (clone HK1.4), anti–Ly6C-pacific blue (PB) (clone HK1.4), Ly6G-Fluorescein isothiocyanate (FITC) (clone 1A8), Ly6G-PerCP-Cy5.5 (clone 1A8), CD3ε-phycoerythrin-cyanine 5 (PeCy5) (clone 145–2C11), Tim4-PE (clone F31-5G3), anti–EpCAM-AF594 (clone G8.8), and anti–CD19-BV650 (clone 6D5).

The following antibodies were purchased from BD: anti–CD11b-Brilliant UltraViolet (BUV) 737 (clone M1/70), purified anti-CD16/32 (clone 2.4G2), and anti–CD24-BUV395 (clone M1/69).

### DT Treatment.

DT (Sigma-Aldrich) was injected intraperitoneally to the mice at a dose of 25 ng/g body weight. PBS was used as a control vehicle. For induction of AKI, mice were injected 24 h later with cisplatin or NaCl.

### Cisplatin-Induced AKI.

AKI was induced in male and female mice by injection of 20 or 15 mg/kg body weight cisplatin, respectively. Results were comparable between male and female mice, although the extent of kidney damage as assessed by blood urea nitrogen and serum creatinine levels varied between mice of different sexes, so that for presentation data were only pooled from mice of the same sex. NaCl was injected as a control for kidney damage. The physical condition of the mice was monitored, and termination criteria were defined using a combination of mouse behavior, physical care condition, movement, body posture, and body weight. When mice reached a weight loss exceeding 20% of the starting weight, the experiment had to be terminated. Blood and kidneys were collected 48 h or 72 h after injection, as indicated. Serum was isolated from blood for creatinine and urea measurements using a Cobas Integra 400 plus analyzer (Roche) and quantified using a cobas c pack CREP2 (Roche) and a cobas c pack UREAL (Roche), respectively.

### Morphologic Evaluation of Kidney Damage.

Kidneys were fixed in 4% formalin in PBS and embedded in paraffin. Sections of 2 μm were cut. Renal lesions were scored on sections stained with Hematoxylin-Eosin and PAS according to standard protocols. Cleaved Caspase 3 immunohistochemistry was performed on a BondRxm system (Leica, Wetzlar, Germany). Briefly, the slides were deparaffinized, and heat-mediated retrieval (ER1) was applied for 20 min. A primary antibody against cleaved Caspase 3 was used (1:150, Cell Signaling, Danvers, cat. No. 9664), and binding was visualized using a Polymer Refine Detection Kit (Leica, Wetzlar, Germany) without postprimary antibody. All slides were scanned with an AT2 scanning system (Leica, Wetzlar, Germany).

Renal tubular injury was scored by the percentage of injured tubules with loss of brush border, cell lysis, and cast formation: 0, no damage; 1, <25%; 2, 25 to 50%; 3, 50 to 75%; 4, >75% (60). Cleaved Caspase 3 positive tubular epithelial cells were counted in 10 high power fields (40×) and averaged.

### Real-Time qPCR.

Total messenger RNA (mRNA) from kidneys was isolated by using RNeasy Midi Kit (Qiagen) according to the manufacturer’s protocol. Complementary DNA was synthesized by using SuperScript II Reverse Transcriptase kit (Invitrogen) according to the manufacturer’s protocol. Real-time polymerase chain reaction (PCR) analysis was performed using SYBR Green Fast master mix (Applied Biosystems) according to the manufacturer’s manual on a StepOnePlus Real-Time PCR Systems (Applied Biosystems) using the relative standard curve method and primers for *Kim-1* (*Kim-1* forward: 5′-CTG​GAA​TGG​CAC​TGT​GAC​ATC​C; *Kim-1* reverse: 5′-GCA​GAT​GCC​AAC​ATA-​GAA​GCC​C) (55) and *Hprt* (*Hprt* forward: 5′-TCA​GTC​AAC​GGG​GGA​CAT​AAA; *Hprt* reverse: 5′-GGG​GCT​GTA​CTG​CTT​AAC​CAG) (7).

### Cytokine Detection.

Cytokines in mouse serum were quantified by flow cytometry using LEGENDplex Mouse Inflammation Panel (Biolegend).

### Statistical Analysis.

Statistical significance was calculated using two-tailed *t* test or Mann–Whitney *U* test (for nonparametric data) in Prism 7 software (GraphPad). Multiple comparison was performed by using one-way ANOVA with Bonferroni correction. A *P* value < 0.05 was considered significant.

## Supplementary Material

Supplementary File

## Data Availability

All study data are included in the article and/or *SI Appendix*.
