# Supplementary material for: Selective depletion of a CD64-expressing phagocyte subset mediates protection against toxic kidney injury and failure
Source: Proc Natl Acad Sci U S A. 2021 Sep 13;118(39):e2022311118. doi: 10.1073/pnas.2022311118 (PMC8488624; doi:10.1073/pnas.2022311118)
Supplement: Supplementary File [file pnas.2022311118.sapp.pdf]

Supplementary Figure 1

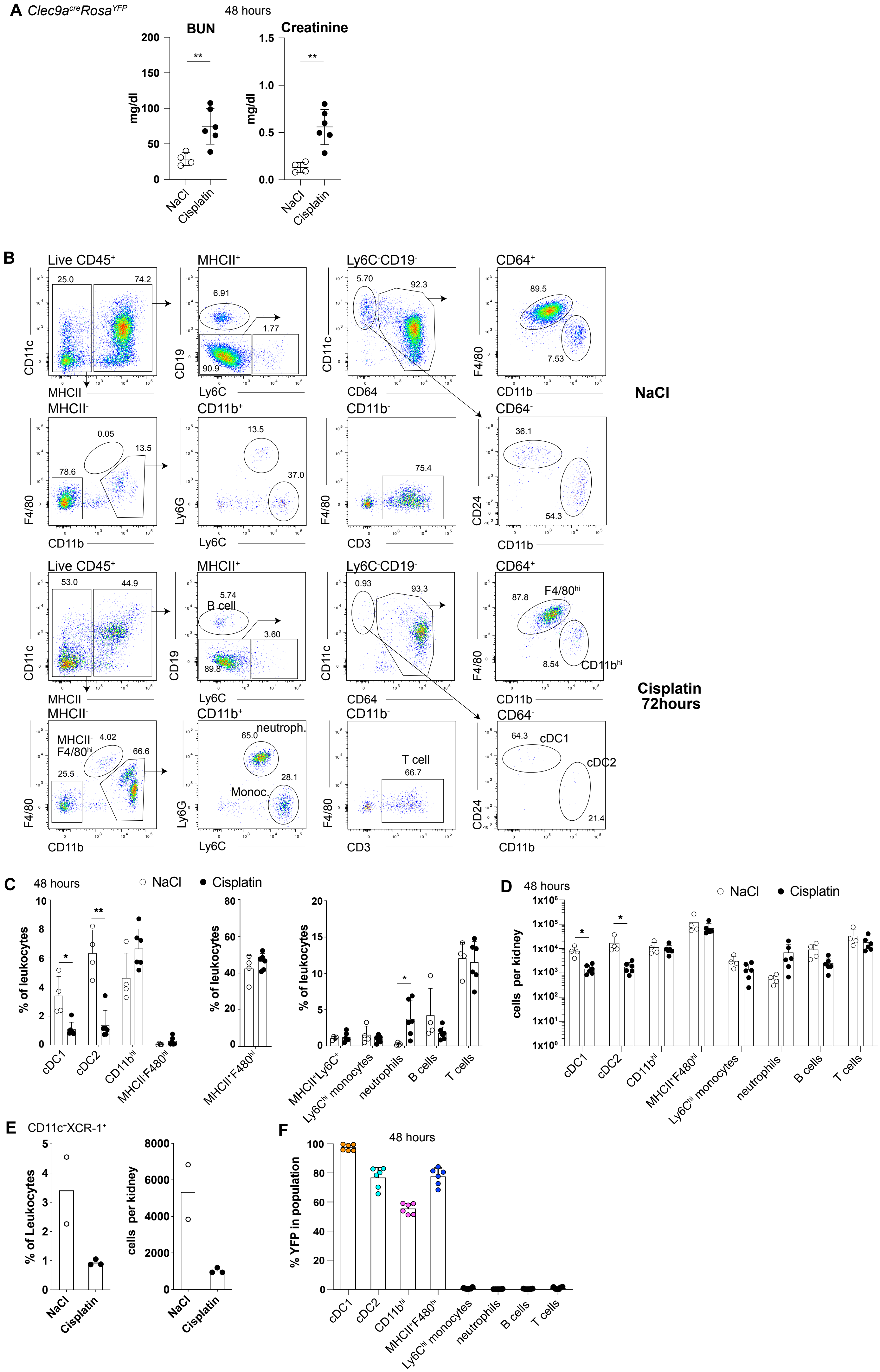

Supplementary Figure legends:

Suppl. Fig. 1. MPs with *Clec9a*-expression history in cisplatin-induced AKI. (A-C) *Clec9a^{cre}Rosa^{YFP}* mice were injected with NaCl or cisplatin and analyzed 48 and 72 hours later. **(A)** Serum creatinine and BUN 48 hours after treatment. **(B)** Representative gating of kidney leukocytes after cisplatin treatment. Live CD45.2^{+} cells were gated as MHCII^{+} and MHCII^{-} cells as indicated. B cells were identified as MHCII^{+}CD19^{+} cells. MHCII^{+}Ly6C^{-}CD19^{-} cells were divided into CD11c^{+}CD64^{-} and CD64^{+} cells. Within CD11c^{+}CD64^{-} cells CD24^{+} cDC1 and CD11b^{+} cDC2 were identified and CD64^{+} cells were further divided into F4/80^{hi} and CD11b^{hi} MPs. MHCII^{-} cells were analyzed for F4/80 and CD11b expression to identify MHCII^{-} F4/80^{hi} MPs and MHCII^{-}CD11b^{+} cells were further divided in Ly6G^{+} neutrophils and Ly6C^{hi} monocytes. CD3^{+} T cells were identified within MHCII^{-}CD11b^{-} gate. **(C, D)** The frequency **(C)** and number **(D)** per kidney of the indicated populations, gated as above, 48 hours after cisplatin treatment. **(E)** Because XCR-1 is a specific marker for cDC1, CD11c^{+}XCR-1^{+} cells were gated without prior exclusion of CD64^{+} cells and quantified 48 hours after cisplatin treatment. **(F)** The percentage of YFP positive cells in the indicated populations 48 after treatment. Each dot represents one mouse. Horizontal bars represent mean, error bars represent SD, * p<0.05, **p<0.01. Data are combined from two independent experiments.

Supplementary Figure 2

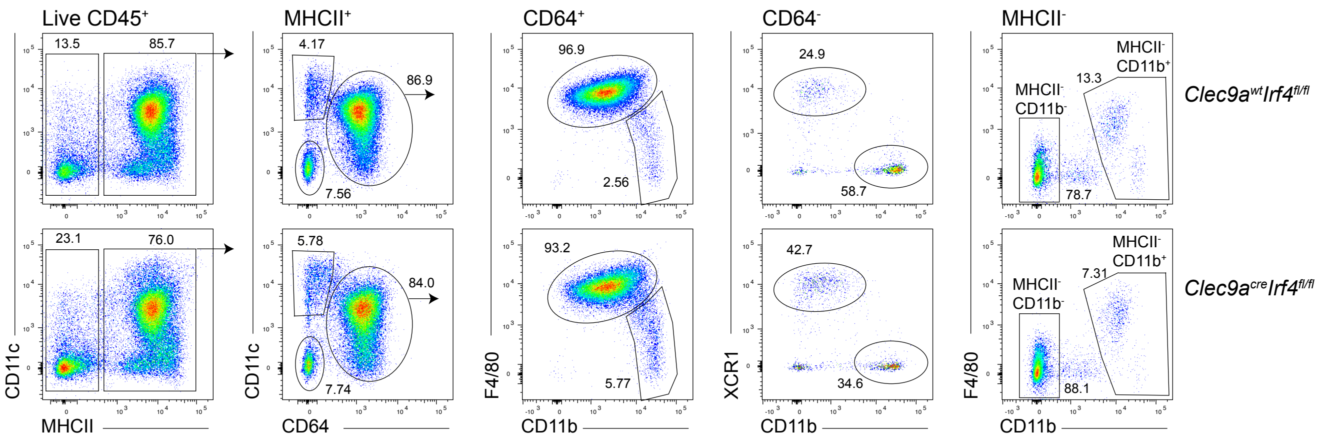

Suppl. Fig. 2. Representative gating strategy of renal leukocytes from *Clec9a*$^{cre/cre}$*Irf4*$^{fl/fl}$ and *Clec9a*$^{wt/wt}$*Irf4*$^{fl/fl}$ mice.

(A) Kidney leukocytes from *Clec9a*$^{cre/cre}$*Irf4*$^{fl/fl}$ and *Clec9a*$^{wt/wt}$*Irf4*$^{fl/fl}$ were analysed by flow cytometry. Live CD45.2^{+}MHCII^{+} cells were gated and divided into CD11c^{+}CD64^{-} and CD64^{+} cells. Within the CD11c^{+}CD64^{-} gate CD24^{+} cDC1 and CD11b^{+} cDC2 were identified and CD64^{+} cells were divided into F4/80^{hi} and CD11b^{hi} MPs. CD11c^{-}CD64^{-} cells were identified as B cells. MHCII^{-} cells were divided into CD11b^{+}, containing mostly neutrophils and monocytes, and CD11b^{-} cells containing mostly T cells (see also Supplementary Figure 1B).

Supplementary Figure 3

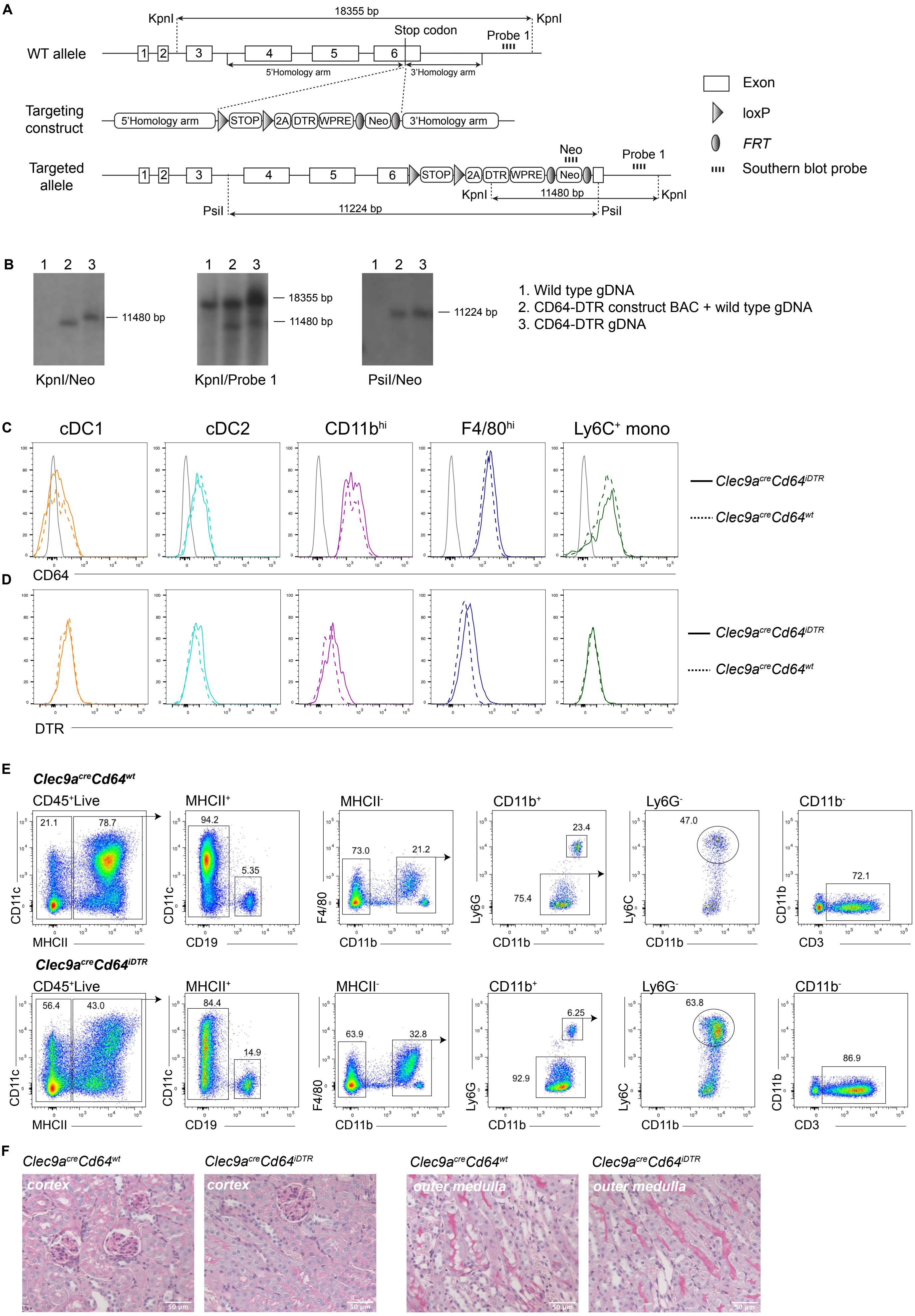

Suppl. Fig. 3. *Clec9a^{cre}Cd64^{iDTR}* mice allow for specific depletion of renal F4/80^{hi} MPs

(A) Schematic representation of targeting the *Cd64* locus. The genomic *Cd64* locus, targeting construct and the targeted allele are shown. Restriction enzyme digestion of the genomic locus with KpnI results in a 18366 bp wild type fragment that is detected by southern blot probe 1. In the KpnI-digested targeted allele probe 1 detects an 18355 bp and an 11480 bp fragment. The 11480 bp fragment is also detected by the Neo probe. Restriction enzyme digestion of the targeted locus with PsiI results in 11224 bp fragment that is detected by the Neo probe. **(B)** Southern blot analysis of the targeted CD64 allele in founder mouse 3. Probe 1 and Neo were used to hybridize KpnI and PsiI digested genomic DNA (gDNA) from founder mouse 3, as well as a wild type mouse as control, as indicated. A Bac containing the CD64-DTR construct mixed with wild type gDNA served as positive control. **(C, D)** Kidney leukocytes from *Clec9a^{cre}Cd64^{iDTR}* and *Clec9a^{cre}Cd64^{wt}* mice were analyzed by flow cytometry. CD64 **(C)** and DTR **(D)** expression on indicated populations from *Clec9a^{cre}Cd64^{iDTR}* and *Clec9a^{cre}Cd64^{wt}* mice are shown. Grey lines in **(C)** represent B cells, which serve as negative control. **(E)** *Clec9a^{cre}Cd64^{iDTR}* and *Clec9a^{cre}Cd64^{wt}* mice were injected with DT and 24 hours later kidneys were analyzed by flow cytometry. Representative gating strategy of renal leukocytes from after DT injection is shown (see also Figure 4B). B cells were identified as CD19^{+}MHCII^{+} cells. Neutrophils and monocytes were identified as MHCII^{-}CD11b^{+}Ly6G^{+} and MHCII^{-}CD11b^{+}Ly6C^{+} cells, respectively. MHCII^{-}CD11b^{-}CD3^{+} cells were identified as T cells. **(F)** Normal kidney architecture in DT-treated *Clec9a$^{wt/cre}$CD64^{iDTR}* mice. Representative PAS staining of renal cortex and outer medulla 24 hours after DT treatment is shown. Scale bar is 50μm.

Supplementary Figure 4

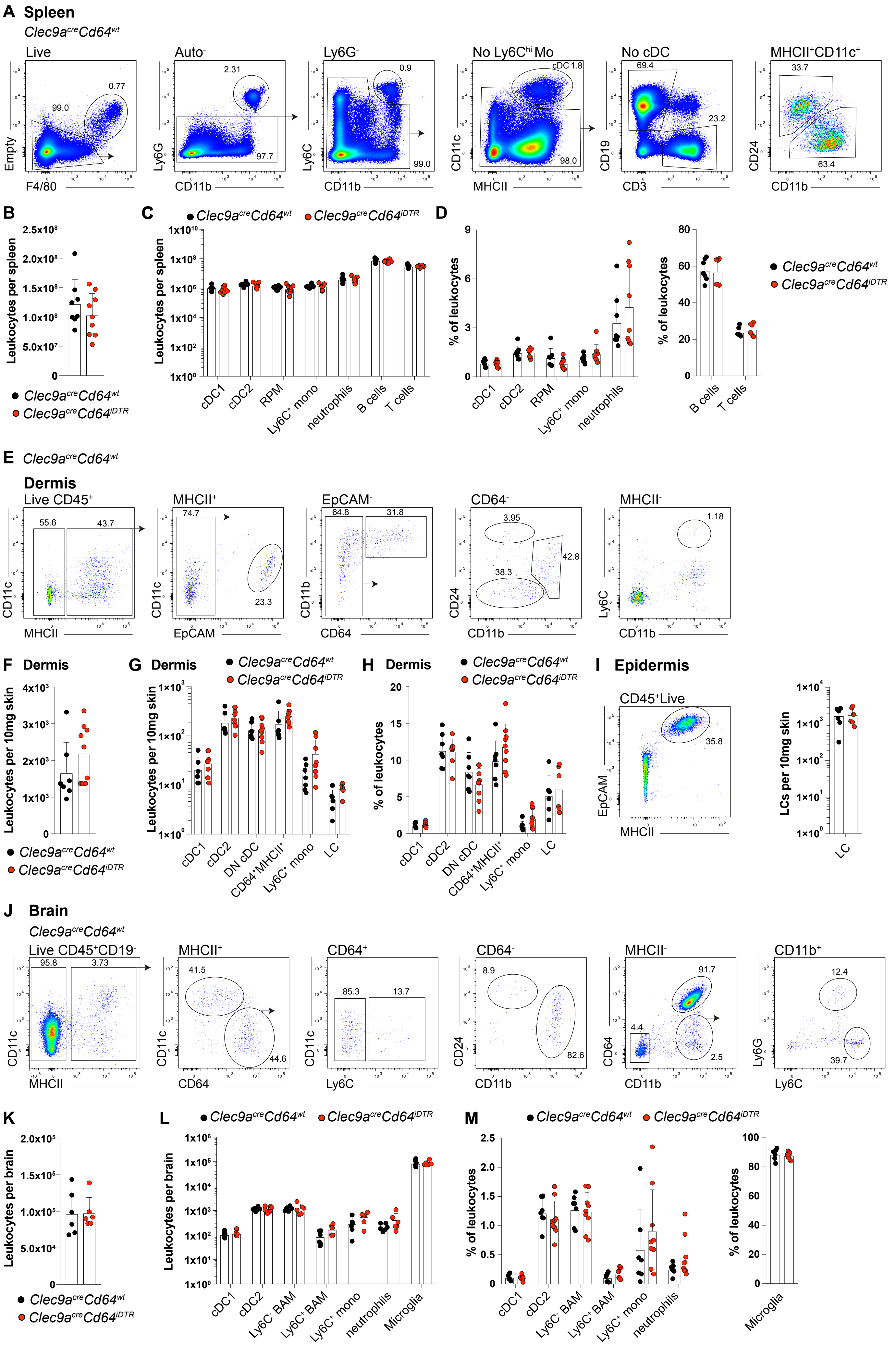

Suppl. Fig 4. No alterations of leukocyte populations in spleen, skin and brain from DT-treated *Clec9a^{cre}Cd64^{iDTR}* and *Clec9a^{cre}Cd64^{wt}* mice

(A-L) *Clec9a^{cre}Cd64^{iDTR}* and *Clec9a^{cre}Cd64^{wt}* mice were treated with DT and 24 hours later were analyzed by flow cytometry. **(A-D)** Spleen leukocytes were analyzed by flow cytometry. **(A)** Single live cells were gated. Autofluorescent F4/80^{hi} cells were identified as red pulp macrophages (RPM). Autofluorescence negative cells were further analyzed for presence of Ly6G^{+}CD11b^{+} neutrophils and Ly6G^{-}Ly6C^{hi}CD11b^{+} monocytes. cDCs were identified as CD11c^{+}MHCII^{+} cells and further subset into CD24^{+} cDC1 and CD11b^{+} cDC2. B and T cells were identified within CD11c^{-} cells, as CD19^{+}CD3^{-} and CD3^{+}CD19^{-} respectively. Total leukocytes per spleen **(B)**, as well as cell number **(C)** and frequency **(D)** of indicated populations per spleen are were quantified. **(E-I)** Ears from DT treated *Clec9a^{cre}Cd64^{iDTR}* and *Clec9a^{cre}Cd64^{wt}* mice were split into dermis and epidermis and leukocytes were isolated for flow cytometry. **(E)** Dermal leukocytes were identified as live CD45.2^{+} cells. Within the MHCII^{+} fraction Langerhans cells (LC) were identified as EpCAM^{+} cells. EpCAM^{-} cells were further divided into CD64^{+} and CD64^{-} cells. Within CD64^{-} cells CD24^{+} cDC1, CD11b^{+} cDC2 and double negative CD24^{-}CD11b^{-} (DN) cDCs were identified. Monocytes were identified as MHCII^{-}CD11b^{+}Ly6C^{hi} cells. Leukocyte populations were quantified relative to skin weight. Total leukocytes **(F)**, as well as the number **(G)** of the indicated populations per 10 mg skin are shown. **(H)** Frequency of indicated populations in dermis. **(I)** LC of the epidermis were identified as CD45^{+}EpCAM^{hi} cells and quantified relative to skin weight. **(J-M)** Brain leukocytes were analyzed by flow cytometry (1). **(J)** Representative gating strategy from *Clec9a^{cre}Cd64^{wt}* mice 24 hours after DT treatment is shown. Live CD45.2^{+} cells were gated and divided into MHCII^{+} and MHCII^{-} cells. MHCII^{+} cells were further divided into CD11c^{+}CD64^{-} and CD64^{+} cells. Within CD11c^{+}CD64^{-} cells CD24^{+} cDC1 and CD11b^{+} cDC2 were identified. CD64^{+} cells were divided in Ly6C^{-} and Ly6C^{+} brain associated macrophages (BAM). Within MHCII^{-} cells microglia were identified as CD11b^{+}CD64^{+} cells, whereas neutrophils and monocytes were defined within CD11b^{+}CD64^{-} cells as Ly6G^{+} and Ly6C^{hi} cells, respectively. Total leukocytes **(K),** as well as cell number **(L)** and frequency **(M)** of indicated populations per brain are shown. Each dot represents one mouse, horizontal bars represent mean and error bars represent SD. Data are combined from at least two independent experiments.

Supplementary Figure 5

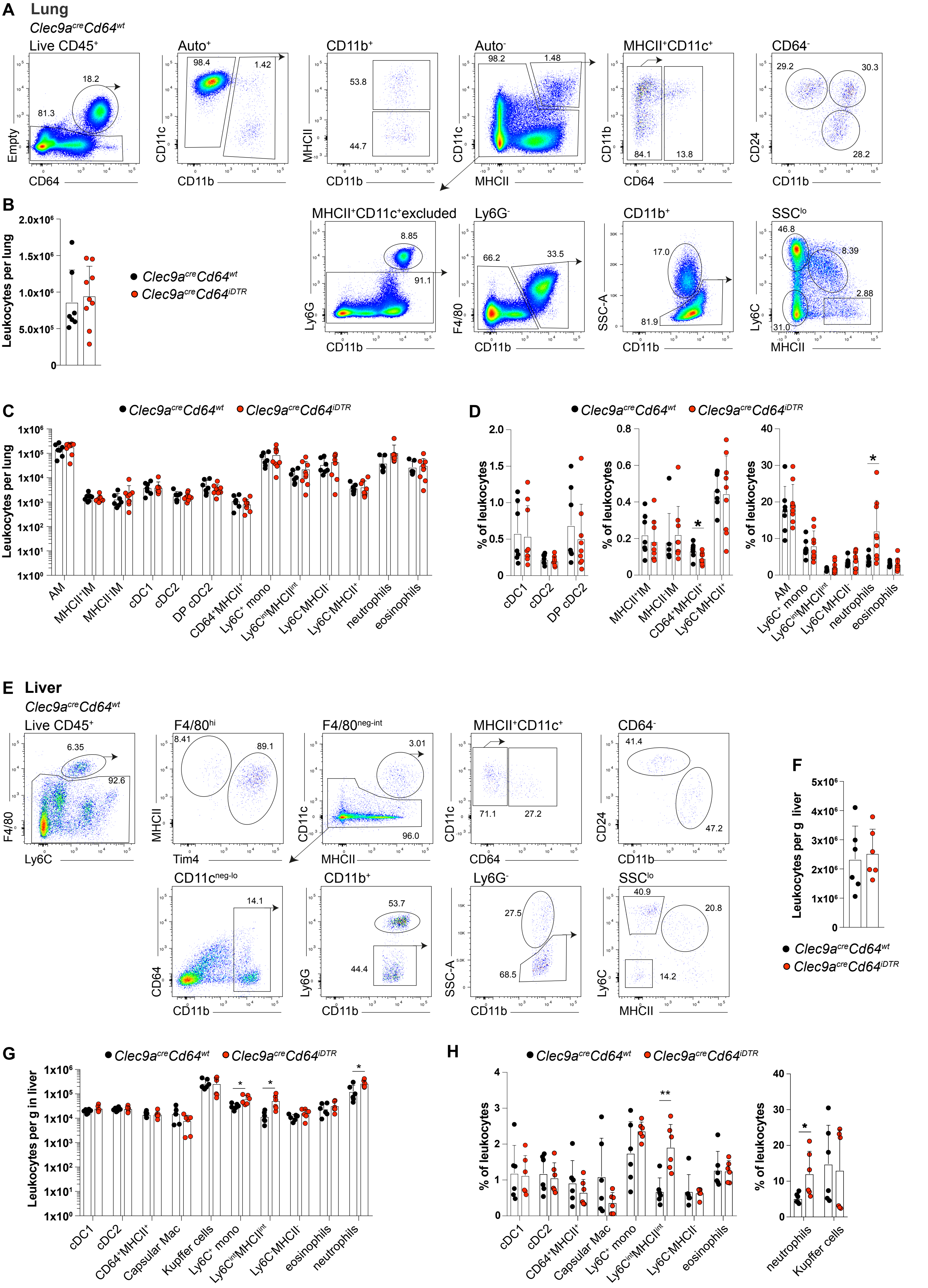

Suppl. Fig 5. No alteration of leukocyte populations in lung and liver of *Clec9a$^{wt/cre}$Cd64^{iDTR}* and *Clec9a$^{wt/cre}$Cd64^{wt}* mice 24 hours after DT treatment.

(A-H) *Clec9a^{cre}Cd64^{iDTR}* and *Clec9a^{cre}Cd64^{wt}* mice were treated with DT and 24 hours later were analyzed by flow cytometry. **(A)** Representative gating strategy of lung leukocytes (2) from *Clec9a^{cre}Cd64^{wt}* mice 24 hours after DT treatment. Leukocytes were identified as live CD45.2^{+} cells. Autofluorescent cells were gated to identify CD11b^{+} alveolar macrophages (AM) and CD11b^{-}MHCII^{+} and CD11b^{-}MHCII^{-} interstitial macrophages (IM). Next, auto^{-} CD11c^{+}MHCII^{+} cells were gated and divided into CD64^{+} monocyte derived cells and CD64^{-} cDCs that were divided into CD24^{+} cDC1, CD11b^{+} cDC2 and double positive (DP) cDC. Within the CD11c low fraction of leukocytes, neutrophils (CD11b^{+}Ly6G^{+}), eosinophils (CD11b^{+} SSC^{hi}) and monocytes (MHCII^{-}CD11b^{+}Ly6C^{hi}) were identified. Total leukocytes **(B)**, as well as number **(C)** and frequency of the indicated populations per lung is shown. Frequency **(D)** of indicated populations in total lung leukocytes is presented. **(E)** Representative gating strategy of liver leukocytes (3) from *Clec9a$^{wt/cre}$CD64^{wt}* mice 24 hours after DT treatment. Within live CD45.2^{+} leukocytes, F4/80^{hi} cells were gated and divided into Tim4^{+} Kupffer cells and Tim4^{-} capsular macrophages (capsular Mac). After exclusion of F4/80^{hi} macrophages, CD11c^{+}MHCII^{+} cells were divided in CD64^{+} monocyte-derived cells and CD64^{-} cells. Within CD64^{-} cells CD24^{+} cDC1 and CD11b^{+} cDC2 were identified. Neutrophils (CD11b^{+}Ly6G^{+} cells), eosinophils (CD11b^{+}SSC^{hi}) and monocytes (MHCII^{-}CD11b^{+}Ly6C^{hi} cells) were gated as indicated. Total leukocytes per gram liver **(F)**, as well as cell number **(G)** and frequency **(H)** of indicated populations are shown. Each dot represents one mouse. Horizontal bars represent mean, error bars represent SD, * $p<0.05$, ** $p<0.01$. Data are combined from at least two independent experiments.

Supplementary Figure 6

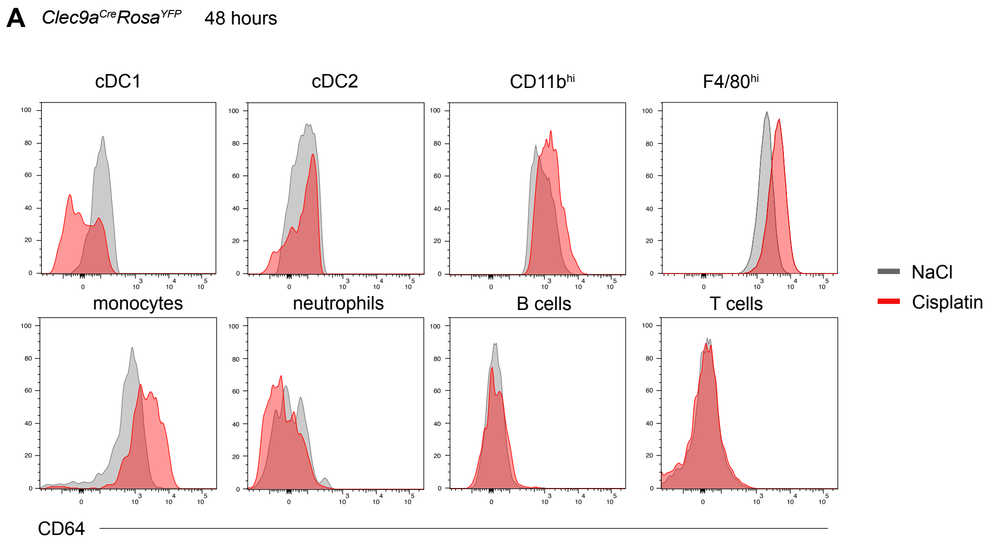

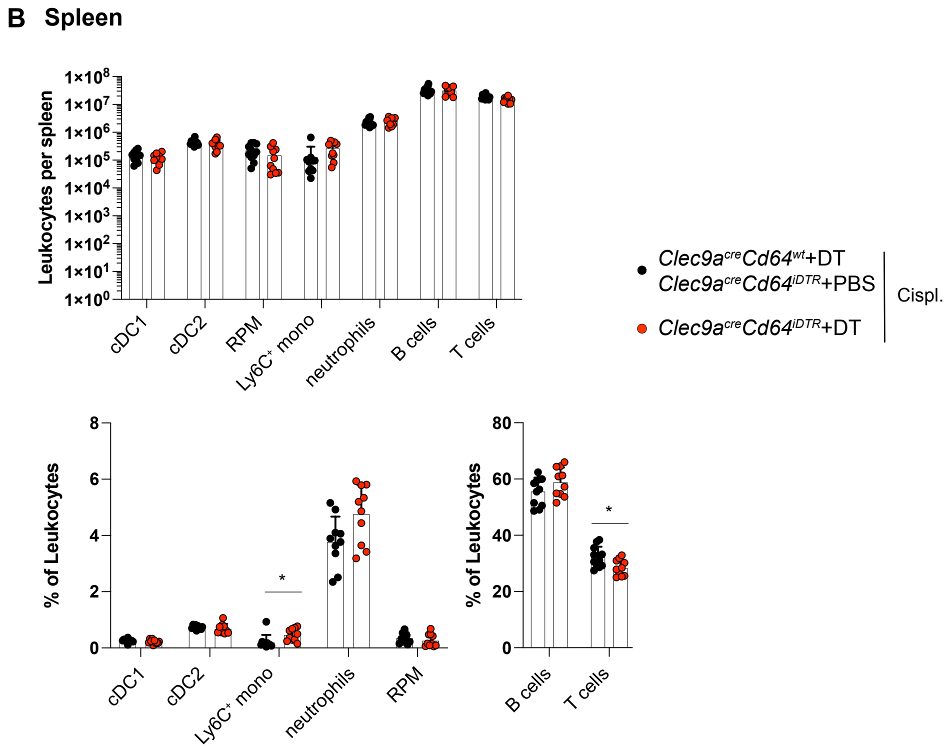

Suppl. Fig 6. Cisplatin does not induce unexpected CD64 expression on kidney leukocytes and *Clec9a*^{cre}*Cd64*^{iDTR} mice show no evidence of systemic neutrophilia in cisplatin AKI. *Clec9a*^{cre}*Cd64*^{iDTR} mice and controls were treated with NaCl or Cisplatin as in Figure 5. **(A)** Kidneys were analyzed by flow cytometry. The indicated populations from control mice treated with NaCl and PBS were gated as in Suppl. Fig. 1B and analyzed for expression of CD64. **(B)** Spleen from cisplatin treated *Clec9a*^{cre}*Cd64*^{iDTR} and control mice were analysed by flow cytometry. The indicated populations were gated and quantified. Each dot represents one mouse. Horizontal bars represent mean, error bars represent SD, * $p<0.05$. Data are combined from at least two independent experiments.

Supplementary Methods:

Cloning of CD64 targeting vector and generation of *Cd64-lox-STOP-lox-DTR* mice

A cassette containing a LoxP – Stop codons – 3x SV40 polyA –LoxP – 2A-DTR-WPRE-bGH polyA- FRT-PGK promoter-Neo-PGK polyA-FRT flanked by two 50 bp arms with homology to the endogenous CD64 locus was cloned into pBlueScript SK II (+) vector by Gibson Assembly. The LoxP – Stop codons – 3x SV40 polyA –LoxP sequence was amplified from Snap25-LSL-2A-GFP targeting vector by PCR, whereby the forward primer was designed to have 50 bp homology with the genomic sequence upstream of endogenous *Cd64* stop codon (Fwd 5' -GGG CGA ATT GGG TAC CTG AAC CCC TTC CTC CCA GTG ACA GTA CTG GGG CAC AAA CTT CCC AAA GTA TAA CTT CGT ATA ATG TAT GCT ATA CGA AG-3'; rev 5' –GCT CCG GAC GGT GCT CCA GAA CCT CT-3'). The 2A-DTR sequence was amplified from GCDL-2A-DTR plasmid (4) (generated by Gunther Kublbeck (DKFZ, Heidelberg) and kindly provided by Natalio Garbi (IEI, Bonn)) using primers: Fwd 5' –GAG CAC CGT CCG GAG CCA CGA ACT TC-3', and Rev 5' –AGG GGG GGC CCG GTA CGT GGG AAT TAG TCA TGC CC-3' . The WPRE – bGH polyA sequence, was amplified from Snap25-LSL-2A-GFP targeting vector (Fwd 5' –GTA CCG GGC CCC CCC GAA ATC ACG TGA GCT TAT C-3', Rev 5' –TTG CGG CCA TCT ATG CCA AGC TTT TAC-3'). The FRT – PGK promoter – Neo – PGK polyA – FRT sequence was amplified from FrtneokanaFrt DTA plasmid by PCR (kindly provided by Markus Moser) by PCR, whereby the reverse primer contained a 50 bp homology to the region downstream of endogenous *Cd64* stop codon. (Fwd 5' –GGC ATA GAT GGC CGC AAG CTT GAT ATC-3', Rev 5' –TCG ATA CCG TCG ACC TAT ATT TGC TTT ATT TAA GAG TTG CAT GCC ATG GTC CCA CAG TTT CAG GGC CTG ACT GAT GAA GTT CC-3'; underlined nucleotides represent homology region). Next, the 3'- and 5'- homology arms were captured by PCR (Fwd 5' –ATG GCG GCC GCA GAC AGA CAT GCA GGC AAA A-3' and Rev 5' –CAT GTC GAC CCA GGA TAA CCC CTG TCC TT-3', Fwd 5' –ATG GTC GAC AAG GCA GGA AAT AGT GTA GCC-3' and Rev 5' –CAT CTC GAG CTG ATG CTG TCT GCT GTG TTC-3') from the RPCIB731E08452Q BAC (BioScience) containing the *Cd64* locus and cloned into pBlueScript SK II (+). The resulting vector was linearized with SalI restriction enzyme, electroporated into EL350 cells and the final CD64 targeting vector was generated by placing the previously assembled cassette, downstream of the 5′ arm and upstream of the 3′ arm, by homologous recombination using EL350 cells. The CD64 targeting vector was linearized using NotI and injected into pronuclei of fertilized C57BL/6N oocytes together with Cas9:crRNA:tracrRNA complex using the following guide RNAs: Fwd AAAGTTGACCCTGAAACTGT and Rev

ACAGTTTCAGGGTCAACTTT. The founder mice were identified by PCR using the following primers: 5′- TGCTCCTGCCGAGAAAGTAT-3′ and 5′- ACAAAGATGTCCCCTTGCAC-3′ (product length 4990 bp, spanning transgene at the 3' end), 5′- GGTACTCTGTTCTCACCCTTC-3′ and 5′- ACCCTCCAAAAACCAAATCC-3′ (product length 6021 bp, spanning transgene at the 5' end), 5′- ACAAAGATGTCCCCTTGCAC-3′ and 5′- CAACAGGTGCTGGCAAGTAA-3′ (product length 1992 bp, endogeneous CD64), 5′- CCCGGTACGTGGGAATTAG-3′ and 5′- TCACGGGCTAAGAGAGGTTC-3′ (product length 760 bp, product within transgene).

Immunofluorescence microscopy

Kidneys were fixed overnight at 4°C in paraformaldehyde, then dehydrated in phosphate buffer containing 30% sucrose overnight at 4°C (5), transferred to Tissue-Tek O.C.T. (Sakura) and frozen on dry ice. 10µm thick frozen sections were cut on a cryostat at -20°C (Leica CM3050S), rehydrated in PBS and permeabilized with Acetone (Sigma-Aldrich). Afterwards the sections were highlighted with a PAP Pen (Kisker Biotech GmbH) and blocked for 1 hour at room temperature (RT) in the dark with blocking buffer (PBS containing 10% goat serum). For staining, antibodies were diluted in blocking buffer and dropped onto sections. Sections were incubated for two hours at RT in the dark with the antibody mixture. Finally, stained sections were washed with PBS, mounted with ProLong™ Diamond Antifade Mountant (Thermo Fisher Scientific), cured at RT for 24 hours in the dark and stored at 4°C until imaging. Confocal microscopy was performed at the Core Facility Bioimaging of the Biomedical Center with an upright Leica SP8X WLL microscope, equipped with 405nm laser, WLL2 laser (470 - 670nm) and acusto-optical beam splitter. Tile-scans were acquired with a 20x0.75 oil objective, pixel size is 1.08um*1.08um, format is 1024*1024, line average is 1, accuracy is 3, frame average is 1, accuracy is 2. The following channel settings were used: DAPI/BV421 (excitation 405nm; emission 415-445nm), AF555 (550; 561-577), AF594 (594; 604-636) and AF647 (647; 658-680). Recording was done sequentially to avoid bleed-through. BV421, AF555, AF594 and AF647 were recorded with hybrid photo detectors, DAPI with a conventional photomultiplier tube. Tile-scans were merged in LAS X (Leica, Version 3.4.1.17670).

The following antibodies were purchased from Biolegend: anti-MHCII I-A/I-E-BV421 (clone M5/114.15.2), anti-CD11b-AF647 (clone M1/70), anti-F4/80-AF594 (clone BM8), anti-F4/80-AF647 (clone BM8). Anti-CD64 (50086-R027, rabbit IgG) was purchased from Sino Biological and goat anti-rabbit IgG-AF555 (1832697) secondary antibody was ordered from Jackson ImmunoResearch.

Supplementary References:

1. Mundt S, et al. (2019) Conventional DCs sample and present myelin antigens in the healthy CNS and allow parenchymal T cell entry to initiate neuroinflammation. *Science Immunology* 4(31):eaau8380.

2. Chakarov S, et al. (2019) Two distinct interstitial macrophage populations coexist across tissues in specific subtissular niches. *Science* 363(6432):eaau0964–14.

3. Sierro F, et al. (2017) A Liver Capsular Network of Monocyte-Derived Macrophages Restricts Hepatic Dissemination of Intraperitoneal Bacteria by Neutrophil Recruitment. *Immunity* 47(2):374–388.e6.

4. Tittel AP, *et al.*, (2012) Functionally relevant neutrophilia in CD11c diphtheria toxin receptor transgenic mice. *Nature Methods* 9, 385–390.

5. M. Bajenoff, N. Glaichenhaus, R. N. Germain, Fibroblastic Reticular Cells Guide T Lymphocyte Entry into and Migration within the Splenic T Cell Zone. *Journal of immunology* **181**, 3947–3954 (2008).
